# Supplementary material for: Self-regulation of socioemotional behavior in twin adolescents: Structural validation of a multidimensional inventory
Source: PLOS Ment Health. 2025 Oct 9;2(10):e0000448. doi: 10.1371/journal.pmen.0000448 (PMC12798259; doi:10.1371/journal.pmen.0000448)
Supplement: S1 Text — (DOCX) [file pmen.0000448.s001.docx]

**S1 Text**

Here we map out the items that make up the MPNI age 17 factors, scales, and subscales that are derived from the current factor analysis (that comprise the MPNI SERI [socioemotional regulation inventory]) with the corresponding items in the MPNI age 12/14 questionnaires to create corresponding factors, scales, and subscales. There are some age-appropriate differences in items from the age 12/14 and age 17 questionnaires.

**MPNI Age 17 Form SERI (Socioemotional Regulation Inventory)**

Self and co-twin ratings

**FACTOR** **I - Low self-regulation, covering externalizing and internalizing problem behaviors** (p-factor)

**Scale: Problem behaviors** (includes subscales of “Hyperactive-inattentive”, “Aggressive behavior” and “Anxious behavior”)

*Subscale: Hyperactive-inattentive*

37 I am hyperactive.

12 I talk all the time.

3 I am restless and can’t sit still.

24 I’m too impatient to wait for my turn.

5 I act before thinking.

7 I am unable to concentrate on anything.

14 I do not listen to directions.

32 I forget things

*Subscale: Aggressive behavior*

33 I often become angry, and I easily get involved in quarrels or fights.

36 When people yell me, I yell back.

11 I sometimes feel the desire to tease, to annoy, or to attach another person without reason.

18 Given enough provocation, I may hit another person.

23 If someone annoys me, I am apt to tell him what I think of him.

15 I spread rumors about other people’s personal matters when I’m mad at them.

8 When I am mad at someone, I sometimes decide to exclude him/her.

*Subscale: Anxious behavior*

9 I usually no not feel at ease when I meet people I do not know too well.

16 I’m scared by or nervous about new things and situations.

27 I am the kind of person who is excessively sensitive and easily hurt.

35 Even though I know I am right, I often have great difficulty getting my points across.

Additional low emotion regulation item that is part of p-factor (but not included in any subscale)

21 My moods change often, and I lose my temper easily.

**FACTOR II - High emotion regulation,** **covering constructive and compliant behaviors (src-factor)**

**Scale: Prosocial behavior**

22 I help others when they need it.

2 I am friendly to others.

17 I’m a person everyone can trust.

6 I try to solve difficult problems reasonably and consider other people.

13 I defend those who are weaker.

10 I sort out things through discussion.

28 I always do my tasks.

4 I am calm and patient.

Additional high emotion regulation item that is part of src-factor (but not included in the prosocial behavior scale)

29 I’m reliable and stable. I keep my composure in all situations.

**MPNI Age 12/14 corresponding to the form SERI Age 17**

Peer (age 12), Parent (age 12), Teacher (age 12 and 14), Self (age 14), Co-twin (age 14) ratings

**FACTOR** **I - Low self-regulation, covering externalizing and internalizing problem behaviors** (p-factor)

**Scale: Problem behaviors** (includes subscales of “Hyperactive-inattentive”, “Aggressive behavior” and “Anxious behavior”)

*Subscale: Hyperactive-inattentive*

(37) Is hyperactive.

(15) Talks all the time.

(4) Is restless and cannot stay put during class.

(29) Is too impatient to wait for s/her turn.

(11) Acts before thinking.

(31) Runs about and climbs everywhere in spite of warnings.

(8) Is unable to concentrate on anything.

(17) Does not listen to directions.

(36) Is forgetful.

*Subscale: Aggressive behavior*

(21) If he/she gets angry at someone, he/she might hit, push, kick, or throw something at hem/her.

(27) Scolds people he/she is upset with.

(13) Teases and attacks another without a reason.

(25) Bullies smaller and weaker kids.

(18) Spreads rumors about other people’s personal matters when he/she is mad at them.

(9) Excludes people from the group by saying, for example, “We don’t want to be with him/her”.

*Subscale: Anxious behavior*

(10) Is shy in front of other students.

(19) Is scared by and nervous about new things or new situations.

(6) Is easily offended/start crying if someone is nasty to him/her

(32) Clings to adults or is too dependent.

Additional item approximating low emotion regulation that is part of p-factor (but not included in any subscales)

(33) Is disobedient at school/home.

**FACTOR II - High emotion regulation,** **covering constructive and compliant behaviors (src-factor)**

**Scale: Prosocial behavior**

(26) Helps others when they need it.

(2) Is friendly to others.

(20) Is a student everyone can trust.

(7) Tries to act reasonably even in difficult situations.

(16) Defends those weaker and smaller.

(12) Sorts things out through discussion.

(34) Is conscientious with homework.

(5) Is patient and calm.

Additional item approximating high emotion regulation that is part of src-factor (but not included in the prosocial behavior scale)

(28) Never quarrels with others.

**Other MPNI items not included in the form SERI factors or subscales above (they loaded on the second factor of the MPNI, activity/passivity)**

*Social status items*

Age 12/14: (1) Is a good leader and would be suitable to lead a class outing.

Age 17: 1 I am a good leader.

Age 12/14: (30) Is popular among his/her friends at school.

Age 17: 26 I’m popular among other youths

*Victimization items*

Age 12/14: (35) Gets teased and taunted a lot.

17: 30 I am often teased.

*Resilience item (not included at age 11/12)*

17: 31 It takes me an unusually long time to get over unpleasant events (reversed)

*Social activity/passivity items*

Age 12/14: (22) Is always together with people during breaks and after school.

Age 17: 19 I’m very energetic, always on the go and other have contact with other people

Age 12/14: (14) Is lonely and has no friends.

Age 17: 34 I am quiet, withdrawn, and often alone

*Items that at age 17 did not present passivity with high self-regulation (compliance) but rather passivity with low self-regulation (anxiety) as seen in the correlations of items and were excluded from the form SERI*

Age 12/14: (23) Avoids difficult situations by doing something else.

Age 17: 20 I avoid difficult situations by doing something else.

Age 17: 25 I give up easily and behave according to expectations

*Depression items (excluded at age 17 from the MPNI because a separate, specific depression scale was administered to the participants); formed an Emotional problems factor with the anxiety items at age 11/12)*

Age 12/14: (3) Seems to be sad and depressed a lot of the time.

Age 12/14: (24) Worries a lot.

*School attendance item (excluded at age 17)*

Age 12/14: (38) Is enthusiastic about school
