# Supplementary material for: Self-regulation of socioemotional behavior in twin adolescents: Structural validation of a multidimensional inventory
Source: PLOS Ment Health. 2025 Oct 9;2(10):e0000448. doi: 10.1371/journal.pmen.0000448 (PMC12798259; doi:10.1371/journal.pmen.0000448)
Supplement: S2 Text — (DOCX) [file pmen.0000448.s002.docx]

**S2 Text**

Social position and activity scale

The reference variables for social activity vs. passivity were not included in any scale for self-regulation of socioemotional behavior because they did not load on the first factor. They did load on the second factor, as well as the items for the participants’ social position (see Others in Table 1). A scale for social position and activity can be formed accordingly from the variables for activity (#19), leadership (#1) and popularity (#26), and (reverse coded) passivity (#34), victimization (#30), giving up (#25), avoidance (#20), and low resilience (#31). Cronbach’s alpha of the scale was 0.65 for males and 0.71 for females.
