# Supplementary material for: Self-regulation of socioemotional behavior in twin adolescents: Structural validation of a multidimensional inventory
Source: PLOS Ment Health. 2025 Oct 9;2(10):e0000448. doi: 10.1371/journal.pmen.0000448 (PMC12798259; doi:10.1371/journal.pmen.0000448)
Supplement: S1 Table — (DOCX) [file pmen.0000448.s004.docx]

**S1 Table**

Tentative results of the main dimensions and of self-regulation of the age 17 MPNI*: Factor analysis, orthogonally rotated factors (N=4105); items grouped according to the components obtained for low and high self-regulation separately.

I II

*Hyperactive/Inattentive behavior*

**37** I am hyperactive 0.50 0.39

**12** I talk all the time 0.40 0.53

**3** I am restless and can’t sit still 0.53 0.14

**24** I’m too impatient to wait for my turn 0.54 0.11

**5**  I act before thinking about 0.42 0.18

**7** I am unable to concentrate on anything 0.45 -0.12

**14** I do not listen to directions 0.50 0.02

**32** I forget things 0.37 -0.08

*Aggressive behavior*

**33** I often become angry, and I easily get involved in quarrels or fights 0.56 0.00

**36** When people yell me, I yell back 0.42 0.26

**11** I sometimes feel the desire to tease, to annoy, or to attach another person

without reason 0.46 0.01

**18** Given enough provocation, I may hit another person 0.39 0.00

**23** If someone annoys me, I am apt to tell him/her what I think of him/her 0.26 0.36

**15** I spread rumors about other people’s personal matter when I am mad at them 0.32 -0.04

**8**  When I am mad at someone, I sometimes decide may exclude him/her 0.33 -0.02

*Anxious behavior*

**9**  I usually do not feel at ease when I meet people I do not know too well 0.18 -0.54

**16** I’m scared by and nervous about new things and situation 0.14 -0.47

**27** I am the kind of person who is excessively sensitive and easily hurt 0.23 -0.34

**35** Even though I know I am right I often have great difficulty getting my points

across 0.16 -0.38

*Emotional self-regulation (reference variables for the two-dimensional model)*

**21** My moods change often, and I lose temper easily 0.55 -0.22

**29** I am reliable and stable. I keep my composure in all situations -0.61 0.19

*Prosocial behavior (constructive and compliant behavior)*

**4** I am calm and patient -0.60 -0.07

**28** I always do my tasks -0.45 0.09

**17** I’m a person everyone can trust -0.35 0.24

**6**  I try to solve difficult problems reasonably and consider other people -0.45 0.25

**2** I am friendly to others -0.35 0.24

**10** I sort out things through discussion -0.26 0.31

**22** I help others when they need it -0.22 0.35

**13** I defend those who are weaker -0.08 0.35

*Social activity (reference variables for the two-dimensional model)*

**19** I’m very energetic, always on the go and often have contact with other people 0.03 0.70

**34** I’m quiet, withdrawn, and often alone -0.03 -0.63

*Others*

1 I am a good leader 0.03 0.51

26 I’m popular among other youths 0.00 0.62

30 I am often teased 0.16 -0.21

31 It takes me an unusually long time to get over unpleasant events 0.27 -0.32

25 I give up easily and behave according to expectations 0.07 -0.28

20 I avoid difficult situations by doing something else 0.13 -0.24

*Items numbered in bold were included in the MPNI Form SERI (Socioemotional Regulation Inventory).
