# Supplementary material for: Self-regulation of socioemotional behavior in twin adolescents: Structural validation of a multidimensional inventory
Source: PLOS Ment Health. 2025 Oct 9;2(10):e0000448. doi: 10.1371/journal.pmen.0000448 (PMC12798259; doi:10.1371/journal.pmen.0000448)
Supplement: S2 Table — (DOCX) [file pmen.0000448.s005.docx]

**S2 Table.** Twin correlations for co-twin ratings at ages 14 and 17

|  | Age 14 | | | | |
| --- | --- | --- | --- | --- | --- |
| Scales/items | MZ M | MZ F | DZ M | DZ F | OS DZ |
| Hyperactive/inattentive | 0.65  [0.53, 0.74] | 0.71  [0.60, 0.79] | 0.40  [0.22, 0.56] | 0.51  [0.33, 0.65] | 0.06  [-0.09, 0.21] |
| Aggressive behavior | 0.57  [0.43, 0.68] | 0.61  [0.48, 0.71] | 0.39  [0.21, 0.55] | 0.59  [0.44, 0.71] | 0.20  [0.05, 0.34] |
| Anxious behavior | 0.61  [0.47, 0.71] | 0.71  [0.61, 0.79] | 0.31  [0.12, 0.49] | 0.49  [0.31, 0.63] | 0.21  [0.06, 0.35] |
| Prosocial behavior | 0.70  [0.59, 0.78] | 0.78  [0.70, 0.85] | 0.22  [0.01, 0.40] | 0.45  [0.27, 0.61] | 0.17  [0.02, 0.31] |
| Low emotion regulation item (#21) | 0.40  [0.27, 0.52] | 0.55  [0.44, 0.65] | 0.06  [-0.11, 0.22] | 0.28  [0.11, 0.44] | -0.04  [-0.17, 0.08] |
| High emotion regulation item (#29) | 0.08  [-0.07, 0.23] | 0.27  [0.13, 0.41] | 0.04  [-0.12, 0.20] | 0.17  [0, 0.34] | 0.06  [-0.06, 0.18] |
|  | Age 17 | | | | |
|  | MZ M | MZ F | DZ M | DZ F | OS DZ |
| Hyperactive/inattentive | 0.39  [0.29, 0.49] | 0.39  [0.3, 0.47] | -0.01  [-0.12, 0.10] | -0.01  [-0.12, 0.10] | -0.02  [-0.1, 0.06] |
| Aggressive behavior | 0.51  [0.42, 0.59] | 0.46  [0.37, 0.54] | 0.24  [0.13, 0.34] | 0.21  [0.1, 0.31] | 0.11  [0.03, 0.19] |
| Anxious behavior | 0.31  [0.2, 0.41] | 0.30  [0.21, 0.39] | 0.01  [-0.1, 0.12] | -0.11  [-0.22, 0] | -0.02  [-0.1, 0.06] |
| Prosocial behavior | 0.44  [0.34, 0.52] | 0.37  [0.28, 0.46] | 0.17  [0.06, 0.28] | 0.19  [0.08, 0.29] | 0.10  [0.03, 0.18] |
| Low emotion regulation item (#21) | 0.29  [0.18, 0.39] | 0.17  [0.07, 0.27] | 0.12  [0.01, 0.22] | 0.05  [-0.06, 0.16] | -0.03  [-0.11, 0.05] |
| High emotion regulation item (#29) | 0.34  [0.24, 0.44] | 0.21  [0.11, 0.3] | 0.08  [-0.03, 0.19] | 0.01  [-0.1, 0.12] | 0.04  [-0.04, 0.12] |
