# Supplementary material for: Self-regulation of socioemotional behavior in twin adolescents: Structural validation of a multidimensional inventory
Source: PLOS Ment Health. 2025 Oct 9;2(10):e0000448. doi: 10.1371/journal.pmen.0000448 (PMC12798259; doi:10.1371/journal.pmen.0000448)
Supplement: S1 Analyses — (DOCX) [file pmen.0000448.s006.docx]

**S1 Analyses**

First, measurement invariance between girls and boys was tested separately for each informant. Because there were cross-loadings across scales, the explorative structural equation model was used. The data at age 14 was weighted due to the enriched data in the intensive sample (i.e., the proportion of enriched data is larger than the true proportion in the population. Therefore, those cases get weights <1, whereas other cases not belonging to the enriched data get a weight of 1. Example: If the proportion of enriched data is two times larger than the true proportion, using 0.5 weights for those cases ends up correcting the proportion in the analysis. By using weights, we can include all cases in the analysis.). Second, measurement invariance across informants was tested separately for girls and boys using confirmatory factor analysis, with only those items that theoretically belonged to a factor taken. Because weighting cannot be used to enriched data at age 14 in this part of invariance tests (i.e., weights are targeted to cases and therefore cannot be used when measures consist of other age measures. For example, in some cases a weight of 0.5 should be used at age 14 but should be 1.0 at other ages. However, using different weights for each age is not possible, thus, a sub-sample of enriched data was randomly chosen that corresponded to a proportion in the population. In all measurement invariance analyses, items were specified as categorical in the model, and the estimator used was the WLSMV in the Mplus statistical program (Muthén, L. K., & Muthén, B. O., 1998–2017). Because twins are more similar than two randomly selected individuals, the standard errors are corrected using the COMPLEX option in Mplus.

Each invariance test is based on hierarchically testing invariance levels with nested models M1–M4 (Putnic & Bornstein, 2016).

M1. Configural invariance – factor loadings and thresholds are freely estimated.

M2. Metric (weak) invariance – factor loadings are set equal, and thresholds are freely estimated.

M3. Scalar (strong) invariance – factor loadings and thresholds are set equal, but residual variances are freely estimated.

M4. Residual (strict) invariance – factor loadings and thresholds are set equal.

For technical reasons, residual variances are set to one in models M1, M2 and M4. Comparing model M1 and M2 gives information about the equality of factor loadings,

comparing M2 and M3 gives information about the equality of thresholds and comparing M3 and M4 gives information about the equality of residual variances.

Model fits were evaluated using the chi-square test, RMSEA, CFI, TLI and SRMR. For a good-fitting model, chi-square test values are non-significant, CFI and TLI are near 0.95 and RMSEA and SRMR are below 0.06 and 0.08, respectively (Hu and Bentler, 2009). Because chi-square difference tests detect minor differences, we used the cut-off values of change in CFI, RMSEA and SRMR given in the article (Chen, F. F., 2007), where the idea is to accept small differences when testing invariance.

As a result, all tested model factor loadings can be set equal. The covariance/correlation structure can be compared between sexes and between informants. Most of the models are also partially invariant when testing thresholds and therefore factor means can be compared between sexes.

Table S1. Measurement invariance test results for hyperactive/inattentive, aggressive behavior, anxious behavior and prosocial factors between boys and girls evaluated by teachers when children are at age 12.

| Model | $\chi^{2}$ | df | CFI | TLI | RMSEA | SRMR | $\Delta$ CFI | $\Delta$ RMSEA | $\Delta$ SRMR |
| --- | --- | --- | --- | --- | --- | --- | --- | --- | --- |
| M1 | 3717.362 | 454 | 0.971 | 0.959 | 0.056 | 0.029 | NA | NA | NA |
| M2 | 2036.481 | 542 | 0.987 | 0.984 | 0.035 | 0.033 | 0.016 | -0.021 | 0.004 |
| M3 | 3069.158 | 590 | 0.978 | 0.976 | 0.043 | 0.034 | -0.005 | 0.003 | 0.001 |
| M4 | 2684.950 | 616 | 0.982 | 0.981 | 0.038 | 0.034 | 0.004 | -0.005 | 0.000 |

Note. M2 model fits the data better than M1 model. Strict invariance holds.

Table S2. Factor correlations, means and variances for hyperactive/inattentive, aggressive behavior, anxious behavior and prosocial factors between boys and girls evaluated by teachers when children are at age 12.

|  | Boys | | | | Girls | | | |
| --- | --- | --- | --- | --- | --- | --- | --- | --- |
|  | Aggressive behavior | Hyperactive/Inattentive | Prosocial | Anxious behavior | Aggressive behavior | Hyperactive/Inattentive | Prosocial | Anxious behavior |
| Aggressive behavior | 1.0 |  |  |  | 1.03 |  |  |  |
| Hyperactive/Inattentive | 0.65 | 1.0 |  |  | 0.65 | 0.83^2^ |  |  |
| Prosocial | -0.10 | -0.46 | 1.0 |  | 0.10^3^ | -0.26^3^ | 0.94 |  |
| Anxious behavior | -0.22 | -0.02 | -0.23 | 1.0 | -0.32^1^ | -0.04 | -0.41^3^ | 1.12 |
| Means | 0 | 0 | 0 | 0 | -0.28^3^ | -0.68^3^ | 0.41^3^ | 0.32^3^ |

Note. Difference between boys and girls ^1^ p < 0.05, ^2^ p < 0.01 and ^3^ p < 0.001.

Table S3. Standardized factor loadings for hyperactive/inattentive, aggressive behavior, anxious behavior and prosocial factors between boys and girls evaluated by teachers when children are at age 12.

|  | Boys | | | | Girls | | | |
| --- | --- | --- | --- | --- | --- | --- | --- | --- |
|  | Aggressive behavior | Hyperactive/Inattentive | Prosocial | Anxious behavior | Aggressive behavior | Hyperactive/Inattentive | Prosocial | Anxious behavior |
| If he/she gets angry at someone, he/she might hit, push, kick, or throw something at hem/her. | **0.647** | 0.098 | -0.382 | -0.008 | **0.697** | 0.095 | -0.394 | -0.009 |
| Scolds people he/she is upset with. | **0.762** | 0.031 | -0.323 | 0.021 | **0.811** | 0.030 | -0.328 | 0.024 |
| Teases and attacks another without a reason. | **0.674** | 0.004 | -0.535 | -0.023 | **0.739** | 0.004 | -0.561 | -0.026 |
| Bullies smaller and weaker kids. | **0.647** | 0.013 | -0.529 | -0.003 | **0.708** | 0.013 | -0.553 | -0.004 |
| Spreads rumors about other people’s personal matters when he/she is mad at them. | **0.706** | 0.033 | -0.179 | 0.206 | **0.737** | 0.031 | -0.178 | 0.225 |
| Excludes people from the group by saying, for example, “We don’t want to be with him/her”. | **0.805** | -0.188 | -0.431 | 0.136 | **0.853** | -0.179 | -0.436 | 0.150 |
| Is hyperactive. | 0.248 | **0.719** | 0.028 | -0.115 | 0.261 | **0.683** | 0.029 | -0.127 |
| Talks all the time. | 0.377 | **0.572** | 0.071 | -0.159 | 0.385 | **0.526** | 0.069 | -0.169 |
| Is restless and cannot stay put during class. | 0.077 | **0.833** | -0.034 | -0.083 | 0.083 | **0.817** | -0.035 | -0.094 |
| Is too impatient to wait for s/her turn. | 0.306 | **0.595** | -0.034 | 0.005 | 0.325 | **0.568** | -0.034 | 0.006 |
| Acts before thinking. | 0.189 | **0.684** | -0.044 | 0.042 | 0.203 | **0.660** | -0.045 | 0.047 |
| Is unable to concentrate on anything. | -0.021 | **0.745** | -0.125 | 0.155 | -0.023 | **0.727** | -0.129 | 0.176 |
| Does not listen to directions. | -0.016 | **0.827** | -0.057 | 0.098 | -0.017 | **0.811** | -0.060 | 0.111 |
| Is forgetful. | -0.428 | **1.037** | 0.024 | 0.084 | -0.464 | **1.011** | 0.024 | 0.095 |
| Is shy in front of other students. | -0.041 | -0.107 | -0.054 | **0.796** | -0.040 | -0.093 | -0.050 | **0.802** |
| Is scared by and nervous about new things or new situations. | 0.059 | 0.032 | 0.090 | **0.791** | 0.059 | 0.028 | 0.085 | **0.822** |
| Is easily offended/start crying if someone is nasty to him/her | 0.459 | -0.037 | 0.023 | **0.505** | 0.469 | -0.034 | 0.023 | **0.540** |
| Clings to adults or is too dependent | 0.193 | 0.249 | 0.148 | **0.606** | 0.196 | 0.228 | 0.144 | **0.646** |
| Helps others when they need it. | 0.051 | 0.014 | **0.836** | -0.016 | 0.052 | 0.013 | **0.817** | -0.017 |
| Is friendly to others. | -0.298 | -0.005 | **0.746** | 0.074 | -0.324 | -0.005 | **0.777** | 0.084 |
| Is a student everyone can trust. | -0.084 | -0.255 | **0.658** | -0.039 | -0.091 | -0.249 | **0.682** | -0.045 |
| Tries to act reasonably even in difficult situations. | 0.122 | -0.487 | **0.479** | -0.139 | 0.131 | -0.469 | **0.489** | -0.155 |
| Defends those weaker and smaller. | 0.059 | 0.104 | **0.843** | 0.036 | 0.059 | 0.095 | **0.816** | 0.038 |
| Sorts things out through discussion. | -0.007 | -0.151 | **0.655** | -0.128 | -0.007 | -0.141 | **0.649** | -0.139 |
| Is conscientious with homework. | 0.515 | -1.011 | **0.137** | 0.000 | 0.569 | -1.006 | **0.144** | 0.000 |
| Is patient and calm. | -0.098 | -0.651 | **0.268** | 0.130 | -0.109 | -0.655 | **0.287** | 0.152 |

Note. Bolded factor loadings belong theoretically to one of the four factors.

Table S4. Measurement invariance test results for hyperactive/inattentive, aggressive behavior, anxious behavior and prosocial factors between boys and girls evaluated by teachers when children are at age 14.

| Model | $\chi^{2}$ | df | CFI | TLI | RMSEA | SRMR | $\Delta$ CFI | $\Delta$ RMSEA | $\Delta$ SRMR |
| --- | --- | --- | --- | --- | --- | --- | --- | --- | --- |
| M1 | 1815.644 | 454 | 0.984 | 0.977 | 0.045 | 0.025 | NA | NA | NA |
| M2 | 1237.507 | 542 | 0.992 | 0.990 | 0.029 | 0.032 | 0.008 | -0.016 | 0.007 |
| M3 | 1585.983 | 590 | 0.988 | 0.987 | 0.033 | 0.031 | -0.004 | 0.004 | 0.001 |
| M4 | 1602.087 | 616 | 0.988 | 0.987 | 0.033 | 0.033 | 0.000 | 0.000 | 0.002 |

Note. M2 model fits the data better than M1 model. Strict invariance holds.

Table S5. Factor correlations, means and variances for hyperactive/inattentive, aggressive behavior, anxious behavior and prosocial factors between boys and girls evaluated by teachers when children are at age 14.

|  | Boys | | | | Girls | | | |
| --- | --- | --- | --- | --- | --- | --- | --- | --- |
|  | Aggressive behavior | Hyperactive/Inattentive | Prosocial | Anxious behavior | Aggressive behavior | Hyperactive/Inattentive | Prosocial | Anxious behavior |
| Aggressive behavior | 1.0 |  |  |  | 1.03 |  |  |  |
| Hyperactive/Inattentive | 0.67 | 1.0 |  |  | 0.75^2^ | 0.96 |  |  |
| Prosocial | -0.11 | 0.41 | 1.0 |  | 0.10^3^ | -0.19^3^ | 1.02 |  |
| Anxious behavior | -0.20 | -0.03 | -0.27 | 1.0 | -0.17 | -0.04 | -0.42^2^ | 1.03 |
| Means | 0 | 0 | 0 | 0 | -0.31^3^ | -0.61^3^ | 0.43^3^ | 0.24^3^ |

Note. Difference between boys and girls ^1^ p < 0.05, ^2^ p < 0.01 and ^3^ p < 0.001.

Table S6. Standardized factor loadings for hyperactive/inattentive, aggressive behavior, anxious behavior and prosocial factors between boys and girls evaluated by teachers when children are at age 14.

|  | Boys | | | | Girls | | | |
| --- | --- | --- | --- | --- | --- | --- | --- | --- |
|  | Aggressive behavior | Hyperactive/Inattentive | Prosocial | Anxious behavior | Aggressive behavior | Hyperactive/Inattentive | Prosocial | Anxious behavior |
| If he/she gets angry at someone, he/she might hit, push, kick, or throw something at hem/her. | **0.679** | 0.139 | -0.224 | 0.054 | **0.704** | 0.139 | -0.232 | 0.056 |
| Scolds people he/she is upset with. | **0.765** | 0.101 | -0.131 | 0.064 | **0.782** | 0.100 | -0.134 | 0.066 |
| Teases and attacks another without a reason. | **0.765** | 0.022 | -0.423 | -0.070 | **0.831** | 0.023 | -0.458 | -0.076 |
| Bullies smaller and weaker kids. | **0.800** | -0.012 | -0.419 | -0.001 | **0.864** | -0.012 | -0.451 | -0.001 |
| Spreads rumors about other people’s personal matters when he/she is mad at them. | **0.614** | 0.220 | 0.000 | 0.249 | **0.611** | 0.211 | 0.000 | 0.247 |
| Excludes people from the group by saying, for example, “We don’t want to be with him/her”. | **0.775** | -0.127 | -0.322 | 0.159 | **0.811** | -0.129 | -0.336 | 0.166 |
| Is hyperactive. | 0.101 | **0.843** | 0.074 | -0.143 | 0.102 | **0.818** | 0.074 | -0.143 |
| Talks all the time. | 0.176 | **0.760** | 0.118 | -0.243 | 0.173 | **0.724** | 0.116 | -0.239 |
| Is restless and cannot stay put during class. | 0.006 | **0.887** | -0.036 | -0.136 | 0.006 | **0.889** | -0.037 | -0.141 |
| Is too impatient to wait for s/her turn. | 0.171 | **0.739** | 0.033 | -0.044 | 0.172 | **0.720** | 0.033 | -0.044 |
| Acts before thinking. | 0.156 | **0.753** | -0.016 | -0.006 | 0.159 | **0.741** | -0.016 | -0.006 |
| Is unable to concentrate on anything. | -0.109 | **0.889** | -0.101 | 0.099 | -0.115 | **0.909** | -0.106 | 0.105 |
| Does not listen to directions. | -0.116 | **0.918** | -0.071 | 0.061 | -0.122 | **0.936** | -0.074 | 0.065 |
| Is forgetful. | -0.349 | **0.952** | -0.009 | 0.173 | -0.372 | **0.981** | -0.010 | 0.183 |
| Is shy in front of other students. | -0.008 | -0.213 | -0.035 | **0.843** | -0.008 | -0.206 | -0.035 | **0.841** |
| Is scared by and nervous about new things or new situations. | 0.075 | 0.011 | 0.124 | **0.886** | 0.077 | 0.010 | 0.126 | **0.899** |
| Is easily offended/start crying if someone is nasty to him/her | 0.444 | -0.018 | 0.209 | **0.636** | 0.445 | -0.018 | 0.208 | **0.636** |
| Clings to adults or is too dependent | 0.263 | 0.161 | 0.189 | **0.671** | 0.264 | 0.157 | 0.189 | **0.671** |
| Helps others when they need it. | -0.052 | -0.018 | **0.820** | -0.051 | -0.053 | -0.018 | **0.827** | -0.051 |
| Is friendly to others. | -0.357 | 0.007 | **0.728** | 0.020 | -0.381 | 0.007 | **0.775** | 0.021 |
| Is a student everyone can trust. | -0.138 | -0.264 | **0.665** | -0.020 | -0.147 | -0.272 | **0.707** | -0.021 |
| Tries to act reasonably even in difficult situations. | 0.104 | -0.489 | **0.503** | -0.084 | 0.111 | -0.503 | **0.532** | -0.090 |
| Defends those weaker and smaller. | -0.015 | 0.126 | **0.877** | 0.046 | -0.015 | 0.121 | **0.867** | 0.046 |
| Sorts things out through discussion. | 0.047 | -0.162 | **0.664** | -0.113 | 0.048 | -0.159 | **0.673** | -0.115 |
| Is conscientious with homework. | 0.383 | -0.906 | **0.245** | 0.002 | 0.420 | -0.961 | **0.267** | 0.002 |
| Is patient and calm. | -0.023 | -0.744 | **0.229** | 0.181 | -0.024 | -0.772 | **0.244** | 0.194 |

Table S7. Measurement invariance test results for hyperactive/inattentive, aggressive behavior, anxious behavior and prosocial factors between boys and girls evaluated by parent(s) when children are at age 12.

| Model | $\chi^{2}$ | df | CFI | TLI | RMSEA | SRMR | $\Delta$ CFI | $\Delta$ RMSEA | $\Delta$ SRMR |
| --- | --- | --- | --- | --- | --- | --- | --- | --- | --- |
| M1 | 2829.023 | 454 | 0.949 | 0.927 | 0.046 | 0.033 | NA | NA | NA |
| M2 | 1963.088 | 541 | 0.970 | 0.964 | 0.033 | 0.036 | 0.021 | -0.013 | 0.003 |
| M3 | 2635.245 | 590 | 0.956 | 0.952 | 0.038 | 0.037 | -0.010 | 0.002 | 0.001 |
| M3^1^ | 2360.466 | 578 | 0.962 | 0.957 | 0.036 | 0.036 | -0.005 | 0.000 | 0.001 |
| M4 | 2505.565 | 616 | 0.960 | 0.957 | 0.035 | 0.037 | 0.004 | -0.003 | 0.000 |
| M4^1^ | 2237.407 | 604 | 0.965 | 0.962 | 0.033 | 0.037 | 0.005 | -0.003 | 0.001 |

Note. M2 model fits the data better than M1 model. ^1^ Partial invariance holds and thresholds for four items were freely estimated; corresponding residual variance is set to one.

Table S8. Factor correlations, means and variances for hyperactive/inattentive, aggressive behavior, anxious behavior and prosocial factors between boys and girls evaluated by parent(s) when children are at age 12.

|  | Boys | | | | Girls | | | |
| --- | --- | --- | --- | --- | --- | --- | --- | --- |
|  | Aggressive behavior | Hyperactive/Inattentive | Prosocial | Anxious behavior | Aggressive behavior | Hyperactive/Inattentive | Prosocial | Anxious behavior |
| Aggressive behavior | 1.0 |  |  |  | 0.95 |  |  |  |
| Hyperactive/Inattentive | 0.52 | 1.0 |  |  | 0.49 | 0.94 |  |  |
| Prosocial | -0.35 | -0.20 | 1.0 |  | -0.35 | -0.18 | 1.12 |  |
| Anxious behavior | 0.21 | 0.10 | -0.08 | 1.0 | 0.21 | 0.09 | -0.18^1^ | 1.00 |
| Means | 0 | 0 | 0 | 0 | 0.07 | -0.49^3^ | 0.42^3^ | 0.09 |

Note. Difference between boys and girls ^1^ p < 0.05, ^2^ p < 0.01 and ^3^ p < 0.001.

Table S9. Standardized factor loadings for hyperactive/inattentive, aggressive behavior, anxious behavior and prosocial factors between boys and girls evaluated by parent(s) when children are at age 12.

|  | Boys | | | | Girls | | | |
| --- | --- | --- | --- | --- | --- | --- | --- | --- |
|  | Aggressive behavior | Hyperactive/Inattentive | Prosocial | Anxious behavior | Aggressive behavior | Hyperactive/Inattentive | Prosocial | Anxious behavior |
| If he/she gets angry at someone, he/she might hit, push, kick, or throw something at hem/her. ^1^ | **0.768** | -0.058 | -0.018 | 0.016 | **0.757** | -0.057 | -0.020 | 0.016 |
| Scolds people he/she is upset with. | **0.723** | -0.023 | 0.009 | -0.013 | **0.713** | -0.023 | 0.009 | -0.013 |
| Teases and attacks another without a reason. ^1^ | **0.667** | 0.076 | -0.069 | 0.058 | **0.659** | 0.075 | -0.074 | 0.059 |
| Bullies smaller and weaker kids. | **0.577** | 0.046 | -0.199 | -0.007 | **0.566** | 0.045 | -0.212 | -0.007 |
| Spreads rumors about other people’s personal matters when he/she is mad at them. | **0.447** | 0.162 | 0.011 | 0.031 | **0.440** | 0.158 | 0.012 | 0.031 |
| Excludes people from the group by saying, for example, “We don’t want to be with him/her”. | **0.531** | 0.017 | -0.053 | 0.080 | **0.521** | 0.017 | -0.056 | 0.080 |
| Is hyperactive. | 0.110 | **0.657** | 0.047 | -0.092 | 0.109 | **0.647** | 0.050 | -0.093 |
| Talks all the time. | 0.343 | **0.438** | 0.174 | -0.200 | 0.337 | **0.429** | 0.186 | -0.202 |
| Is restless and cannot stay put during class. | 0.018 | **0.807** | 0.011 | 0.004 | 0.018 | **0.799** | 0.012 | 0.005 |
| Is too impatient to wait for s/her turn. | 0.228 | **0.520** | -0.002 | 0.020 | 0.226 | **0.513** | -0.002 | 0.021 |
| Acts before thinking. | 0.218 | **0.524** | -0.025 | 0.037 | 0.216 | **0.517** | -0.027 | 0.038 |
| Is unable to concentrate on anything. | -0.014 | **0.755** | -0.010 | 0.179 | -0.014 | **0.745** | -0.011 | 0.183 |
| Does not listen to directions. | 0.172 | **0.556** | -0.046 | 0.062 | 0.170 | **0.547** | -0.050 | 0.063 |
| Is forgetful. | -0.032 | **0.517** | -0.065 | 0.143 | -0.032 | **0.505** | -0.069 | 0.144 |
| Is shy in front of other students. | 0.016 | -0.108 | -0.063 | **0.703** | 0.015 | -0.104 | -0.066 | **0.700** |
| Is scared by and nervous about new things or new situations. | 0.019 | 0.031 | 0.055 | **0.702** | 0.019 | 0.031 | 0.059 | **0.706** |
| Is easily offended/start crying if someone is nasty to him/her | 0.231 | 0.026 | 0.178 | **0.364** | 0.226 | 0.026 | 0.190 | **0.368** |
| Clings to adults or is too dependent | 0.034 | 0.203 | 0.045 | **0.578** | 0.034 | 0.198 | 0.048 | **0.582** |
| Helps others when they need it. | -0.021 | 0.076 | **0.776** | -0.012 | -0.020 | 0.071 | **0.792** | -0.012 |
| Is friendly to others. | -0.246 | -0.016 | **0.563** | 0.098 | -0.236 | -0.015 | **0.589** | 0.097 |
| Is a student everyone can trust. | -0.095 | -0.253 | **0.571** | 0.039 | -0.091 | -0.242 | **0.598** | 0.038 |
| Tries to act reasonably even in difficult situations. ^1^ | 0.118 | -0.364 | **0.598** | -0.071 | 0.113 | -0.346 | **0.621** | -0.070 |
| Defends those weaker and smaller. | -0.023 | 0.150 | **0.749** | 0.005 | -0.022 | 0.140 | **0.767** | 0.005 |
| Sorts things out through discussion. | -0.019 | -0.186 | **0.571** | -0.063 | -0.019 | -0.177 | **0.594** | -0.062 |
| Is conscientious with homework. | 0.167 | -0.524 | **0.375** | 0.027 | 0.163 | -0.508 | **0.397** | 0.027 |
| Is patient and calm. ^1^ | -0.023 | -0.570 | **0.309** | 0.145 | -0.023 | -0.559 | **0.332** | 0.147 |

Note. ^1^ Thresholds cannot be set equal between boys and girls.

Table S10. Measurement invariance test results for hyperactive/inattentive, aggressive behavior, anxious behavior and prosocial factors between boys and girls evaluated by self when children are at age 14.

| Model | $\chi^{2}$ | df | CFI | TLI | RMSEA | SRMR | $\Delta$ CFI | $\Delta$ RMSEA | $\Delta$ SRMR |
| --- | --- | --- | --- | --- | --- | --- | --- | --- | --- |
| M1 | 1010.661 | 454 | 0.962 | 0.945 | 0.037 | 0.034 | NA | NA | NA |
| M2 | 976.785 | 541 | 0.970 | 0.964 | 0.030 | 0.041 | 0.008 | -0.007 | 0.007 |
| M3 | 1188.278 | 588 | 0.959 | 0.954 | 0.033 | 0.043 | -0.010 | 0.002 | 0.003 |
| M3^1^ | 1220.722 | 583 | 0.963 | 0.959 | 0.033 | 0.042 | -0.005 | 0.000 | 0.001 |
| M4 | 1193.899 | 614 | 0.960 | 0.958 | 0.032 | 0.044 | 0.001 | -0.001 | 0.001 |
| M4^1^ | 1117.934 | 608 | 0.965 | 0.963 | 0.030 | 0.042 | 0.002 | -0.003 | 0.000 |

Note. M2 model fits the data better than M1 model. ^1^ Partial invariance holds and thresholds for two items were freely estimated; corresponding residual variance is set to one.

Table S11. Factor correlations, means and variances results for hyperactive/inattentive, aggressive behavior, anxious behavior and prosocial factors between boys and girls evaluated by self when children are at age 14.

|  | Boys | | | | Girls | | | |
| --- | --- | --- | --- | --- | --- | --- | --- | --- |
|  | Aggressive behavior | Hyperactive/Inattentive | Prosocial | Anxious behavior | Aggressive behavior | Hyperactive/Inattentive | Prosocial | Anxious behavior |
| Aggressive behavior | 1.0 |  |  |  | 1.17 |  |  |  |
| Hyperactive/Inattentive | 0.58 | 1.0 |  |  | 0.45^2^ | 1.12 |  |  |
| Prosocial | -0.26 | -0.27 | 1.0 |  | -0.24 | -0.30 | 1.02 |  |
| Anxious behavior | 0.26 | 0.10 | 0.06 | 1.0 | 0.11 | -0.05^1^ | -0.23^3^ | 1.08 |
| Means | 0 | 0 | 0 | 0 | -0.74^3^ | -0.07 | 0.52^3^ | 0.44^3^ |

Note. Difference between boys and girls ^1^ p < 0.05, ^2^ p < 0.01 and ^3^ p < 0.001.

Table S12. Standardized factor loadings results for hyperactive/inattentive, aggressive behavior, anxious behavior and prosocial factors between boys and girls evaluated by self when children are at age 14.

|  | Boys | | | | Girls | | | |
| --- | --- | --- | --- | --- | --- | --- | --- | --- |
|  | Aggressive behavior | Hyperactive/Inattentive | Prosocial | Anxious behavior | Aggressive behavior | Hyperactive/Inattentive | Prosocial | Anxious behavior |
| I often become angry, and I easily get involved in quarrels or fights | **0.693** | -0.036 | -0.079 | 0.027 | **0.720** | -0.037 | -0.077 | 0.027 |
| When people yell me, I yell back. | **0.589** | 0.001 | 0.037 | 0.158 | **0.626** | 0.001 | 0.037 | 0.162 |
| I sometimes feel the desire to tease, to annoy, or to attach another person without reason. | **0.731** | 0.020 | 0.008 | -0.029 | **0.755** | 0.020 | 0.008 | -0.028 |
| Given enough provocation, I may hit another person | **0.668** | 0.064 | -0.089 | -0.057 | **0.694** | 0.066 | -0.087 | -0.057 |
| I spread rumors about other people’s personal matter when I am mad at them. | **0.522** | 0.028 | 0.024 | 0.114 | **0.557** | 0.030 | 0.024 | 0.117 |
| When I am mad at someone, I sometimes decide may exclude him/her. | **0.444** | 0.036 | 0.026 | 0.123 | **0.476** | 0.038 | 0.026 | 0.127 |
| I am hyperactive. | 0.011 | **0.702** | 0.250 | -0.045 | 0.012 | **0.722** | 0.246 | -0.045 |
| I Talk all the time. | 0.010 | **0.668** | 0.320 | -0.179 | 0.011 | **0.671** | 0.307 | -0.176 |
| I am restless and can’t sit still. | 0.051 | **0.711** | 0.047 | 0.053 | 0.054 | **0.738** | 0.047 | 0.054 |
| I’m too impatient to wait for my turn. | 0.113 | **0.503** | -0.025 | 0.097 | 0.121 | **0.529** | -0.025 | 0.100 |
| I act before thinking about. | -0.009 | **0.492** | 0.140 | 0.031 | -0.009 | **0.516** | 0.140 | 0.032 |
| I am unable to concentrate on anything. | 0.019 | **0.593** | -0.093 | 0.233 | 0.020 | **0.621** | -0.093 | 0.239 |
| I do not listen to directions. | 0.170 | **0.518** | -0.091 | 0.093 | 0.182 | **0.544** | -0.092 | 0.095 |
| I forget things. | 0.041 | **0.468** | -0.018 | 0.224 | 0.044 | **0.496** | -0.019 | 0.232 |
| I usually do not feel at ease when I meet people I do not know too well. | -0.016 | -0.065 | -0.089 | **0.609** | -0.017 | -0.066 | -0.087 | **0.609** |
| I’m scared by and nervous about new things and situation. | -0.009 | 0.056 | 0.093 | **0.629** | -0.010 | 0.060 | 0.095 | **0.657** |
| I am the kind of person who is excessively sensitive and easily hurt.^1^ | 0.162 | -0.028 | 0.157 | **0.473** | 0.179 | -0.030 | 0.162 | **0.501** |
| Even though I know I am right I often have great difficulty getting my points across | 0.189 | 0.076 | 0.194 | **0.250** | 0.208 | 0.082 | 0.200 | **0.265** |
| I help others when they need it. | -0.014 | 0.020 | **0.765** | 0.071 | -0.015 | 0.021 | **0.782** | 0.074 |
| I am friendly to others. | -0.097 | -0.152 | **0.589** | -0.021 | -0.103 | -0.158 | **0.589** | -0.021 |
| I’m a person everyone can trust. | -0.051 | -0.151 | **0.567** | 0.028 | -0.055 | -0.159 | **0.571** | 0.029 |
| I try to solve difficult problems reasonably and consider other people. | 0.145 | -0.268 | **0.564** | -0.118 | 0.151 | -0.273 | **0.550** | -0.118 |
| I’m a person everyone can trust. | -0.061 | 0.176 | **0.670** | 0.035 | -0.066 | 0.187 | **0.681** | 0.036 |
| I sort out things through discussion. | -0.057 | -0.139 | **0.507** | -0.017 | -0.061 | -0.146 | **0.508** | -0.017 |
| I always do my tasks. | 0.030 | -0.491 | **0.312** | 0.026 | 0.032 | -0.508 | **0.309** | 0.026 |
| I am calm and patient.^1^ | 0.027 | -0.599 | **0.281** | 0.104 | 0.028 | -0.614 | **0.275** | 0.105 |

Table S13. Measurement invariance test results for hyperactive/inattentive, aggressive behavior, anxious behavior and prosocial factors between boys and girls evaluated by their co-twin when children are at age 14.

| Model | $\chi^{2}$ | df | CFI | TLI | RMSEA | SRMR | $\Delta$ CFI | $\Delta$ RMSEA | $\Delta$ SRMR |
| --- | --- | --- | --- | --- | --- | --- | --- | --- | --- |
| M1 | 1304.201 | 454 | 0.966 | 0.952 | 0.045 | 0.031 | NA | NA | NA |
| M2 | 1086.734 | 541 | 0.978 | 0.973 | 0.033 | 0.037 | 0.012 | -0.012 | 0.006 |
| M3 | 1370.426 | 590 | 0.968 | 0.965 | 0.038 | 0.037 | 0.008 | 0.002 | 0.002 |
| M4 | 1361.128 | 616 | 0.970 | 0.968 | 0.036 | 0.039 | 0.002 | -0.002 | 0.002 |

Note. M2 model fits the data better than M1 model. Strict invariance holds.

Table S14. Factor correlations, means and variances for hyperactive/inattentive, aggressive behavior, anxious behavior and prosocial factors between boys and girls evaluated by their co-twin when children are at age 14.

|  | Boys | | | | Girls | | | |
| --- | --- | --- | --- | --- | --- | --- | --- | --- |
|  | Aggressive behavior | Hyperactive/Inattentive | Prosocial | Anxious behavior | Aggressive behavior | Hyperactive/Inattentive | Prosocial | Anxious behavior |
| Aggressive behavior | 1.0 |  |  |  | 1.04 |  |  |  |
| Hyperactive/Inattentive | 0.56 | 1.0 |  |  | 0.60 | 1.10 |  |  |
| Prosocial | -0.26 | -0.28 | 1.0 |  | -0.39^1^ | -0.39^1^ | 0.99 |  |
| Anxious behavior | 0.24 | 0.08 | -0.01 | 1.0 | 0.17 | -0.02 | -0.04 | 0.78^1^ |
| Means | 0 | 0 | 0 | 0 | -0.53^3^ | -0.39^3^ | 0.64^3^ | 0.44^3^ |

Note. Difference between boys and girls ^1^ p < 0.05, ^2^ p < 0.01 and ^3^ p < 0.001.

Table S15. Standardized factor loadings for hyperactive/inattentive, aggressive behavior, anxious behavior and prosocial factors between boys and girls evaluated by their co-twin when children are at age 14.

|  | Boys | | | | Girls | | | |
| --- | --- | --- | --- | --- | --- | --- | --- | --- |
|  | Aggressive behavior | Hyperactive/Inattentive | Prosocial | Anxious behavior | Aggressive behavior | Hyperactive/Inattentive | Prosocial | Anxious behavior |
| I often become angry, and I easily get involved in quarrels or fights | **0.749** | -0.023 | -0.021 | -0.013 | **0.754** | -0.024 | -0.020 | -0.011 |
| When people yell me, I yell back. | **0.653** | 0.011 | 0.042 | 0.105 | **0.667** | 0.011 | 0.042 | 0.093 |
| I sometimes feel the desire to tease, to annoy, or to attach another person without reason. | **0.798** | 0.063 | -0.010 | -0.006 | **0.799** | 0.065 | -0.010 | -0.005 |
| Given enough provocation, I may hit another person | **0.707** | 0.089 | -0.055 | -0.003 | **0.705** | 0.092 | -0.054 | -0.003 |
| I spread rumors about other people’s personal matter when I am mad at them. | **0.667** | -0.029 | 0.015 | 0.215 | **0.688** | -0.031 | 0.016 | 0.192 |
| When I am mad at someone, I sometimes decide may exclude him/her. | **0.601** | 0.017 | 0.023 | 0.162 | **0.616** | 0.018 | 0.023 | 0.144 |
| I am hyperactive. | 0.022 | **0.762** | 0.229 | -0.040 | 0.022 | **0.791** | 0.225 | -0.035 |
| I Talk all the time. | 0.031 | **0.672** | 0.269 | -0.105 | 0.032 | **0.700** | 0.266 | -0.092 |
| I am restless and can’t sit still. | -0.054 | **0.867** | 0.057 | -0.026 | -0.053 | **0.884** | 0.055 | -0.023 |
| I’m too impatient to wait for my turn. | 0.134 | **0.583** | -0.009 | 0.121 | 0.134 | **0.603** | -0.009 | 0.106 |
| I act before thinking about. | 0.065 | **0.604** | 0.011 | 0.075 | 0.065 | **0.624** | 0.010 | 0.065 |
| I am unable to concentrate on anything. | 0.019 | **0.747** | -0.088 | 0.163 | 0.019 | **0.767** | -0.086 | 0.141 |
| I do not listen to directions. | 0.061 | **0.705** | -0.087 | 0.091 | 0.060 | **0.719** | -0.085 | 0.078 |
| I forget things. | -0.023 | **0.558** | -0.009 | 0.202 | -0.023 | **0.586** | -0.009 | 0.179 |
| I usually do not feel at ease when I meet people I do not know too well. | 0.002 | -0.129 | -0.128 | **0.692** | 0.002 | -0.142 | -0.133 | **0.639** |
| I’m scared by and nervous about new things and situation. | -0.069 | 0.031 | 0.021 | **0.711** | -0.074 | 0.034 | 0.022 | **0.665** |
| I am the kind of person who is excessively sensitive and easily hurt. | 0.148 | 0.006 | 0.157 | **0.572** | 0.159 | 0.007 | 0.164 | **0.532** |
| Even though I know I am right I often have great difficulty getting my points across | 0.110 | 0.117 | 0.090 | **0.393** | 0.115 | 0.127 | 0.092 | **0.357** |
| I help others when they need it. | -0.161 | 0.040 | **0.730** | 0.026 | -0.162 | 0.041 | **0.720** | 0.023 |
| I am friendly to others. | -0.295 | -0.095 | **0.543** | 0.014 | -0.291 | -0.097 | **0.524** | 0.012 |
| I’m a person everyone can trust. | -0.166 | -0.153 | **0.587** | -0.024 | -0.165 | -0.157 | **0.570** | -0.020 |
| I try to solve difficult problems reasonably and consider other people. | 0.055 | -0.289 | **0.629** | -0.098 | 0.055 | -0.299 | **0.615** | -0.085 |
| I’m a person everyone can trust. | -0.260 | 0.182 | **0.631** | 0.046 | -0.264 | 0.191 | **0.625** | 0.041 |
| I sort out things through discussion. | -0.132 | -0.149 | **0.560** | -0.024 | -0.132 | -0.153 | **0.546** | -0.021 |
| I always do my tasks. | 0.025 | -0.468 | **0.359** | 0.055 | 0.025 | -0.477 | **0.347** | 0.047 |
| I am calm and patient. | 0.021 | -0.547 | **0.326** | 0.084 | 0.021 | -0.554 | **0.313** | 0.071 |

Table S16. Measurement invariance test results for hyperactive/inattentive, aggressive behavior, anxious behavior and prosocial factors between boys and girls evaluated by themselves when children are at age 17.

| Model | $\chi^{2}$ | df | CFI | TLI | RMSEA | SRMR | $\Delta$ CFI | $\Delta$ RMSEA | $\Delta$ SRMR |
| --- | --- | --- | --- | --- | --- | --- | --- | --- | --- |
| M1 | 1913.140 | 454 | 0.955 | 0.935 | 0.039 | 0.029 | NA | NA | NA |
| M2 | 1640.284 | 542 | 0.966 | 0.959 | 0.031 | 0.033 | 0.011 | -0.008 | 0.04 |
| M3 | 2466.474 | 589 | 0.942 | 0.936 | 0.039 | 0.036 | -0.024 | 0.007 | 0.003 |
| M3^1^ | 1833.990 | 571 | 0.961 | 0.955 | 0.032 | 0.033 | -0.003 | 0.000 | 0.001 |
| M4 | 2493.463 | 615 | 0.942 | 0.938 | 0.038 | 0.036 | 0.000 | -0.001 | 0.003 |
| M4^1^ | 1791.462 | 597 | 0.963 | 0.960 | 0.031 | 0.034 | 0.002 | -0.001 | 0.001 |

Note. M2 model fits the data better than M1 model. ^1^ Partial invariance holds and thresholds for five items were freely estimated; corresponding residual variance is set to one.

Table S17. Factor correlations, means and variances for hyperactive/inattentive, aggressive behavior, anxious behavior and prosocial factors between boys and girls evaluated by themselves when children are at age 17.

|  | Boys | | | | Girls | | | |
| --- | --- | --- | --- | --- | --- | --- | --- | --- |
|  | Aggressive behavior | Hyperactive/Inattentive | Prosocial | Anxious behavior | Aggressive behavior | Hyperactive/Inattentive | Prosocial | Anxious behavior |
| Aggressive behavior | 1.0 |  |  |  | 1.01 |  |  |  |
| Hyperactive/Inattentive | 0.60 | 1.0 |  |  | 0.57 | 1.14^1^ |  |  |
| Prosocial | -0.17 | -0.12 | 1.0 |  | -0.22 | -0.13 | 1.02 |  |
| Anxious behavior | 0.01 | -0.01 | -0.18 | 1.0 | -0.5 | -0.15^2^ | -0.20 | 1.13 |
| Means | 0 | 0 | 0 | 0 | 0.17^3^ | 0.08 | 0.42^3^ | 0.31^3^ |

Note. Difference between boys and girls ^1^ p < 0.05, ^2^ p < 0.01 and ^3^ p < 0.001.

Table S18. Standardized factor loadings for hyperactive/inattentive, aggressive behavior, anxious behavior and prosocial factors between boys and girls evaluated by themselves when children are at age 17.

|  | Boys | | | | Girls | | | |
| --- | --- | --- | --- | --- | --- | --- | --- | --- |
|  | Aggressive behavior | Hyperactive/Inattentive | Prosocial | Anxious behavior | Aggressive behavior | Hyperactive/Inattentive | Prosocial | Anxious behavior |
| I often become angry, and I easily get involved in quarrels or fights | **0.710** | 0.067 | -0.042 | 0.016 | **0.709** | 0.071 | -0.042 | 0.017 |
| When people yell me, I yell back. | **0.581** | 0.037 | 0.130 | -0.196 | **0.584** | 0.040 | 0.132 | -0.209 |
| I sometimes feel the desire to tease, to annoy, or to attach another person without reason. | **0.417** | 0.155 | -0.065 | 0.050 | **0.416** | 0.165 | -0.065 | 0.053 |
| Given enough provocation, I may hit another person ^1^ | **0.753** | -0.147 | -0.013 | -0.015 | **0.754** | 0.156 | -0.013 | -0.016 |
| I spread rumors about other people’s personal matter when I am mad at them. | **0.363** | 0.113 | -0.084 | 0.137 | **0.363** | 0.120 | -0.084 | 0.146 |
| When I am mad at someone, I sometimes decide may exclude him/her. | **0.458** | 0.018 | 0.008 | 0.080 | **0.459** | 0.019 | 0.008 | 0.084 |
| I am hyperactive. | 0.037 | **0.682** | 0.142 | -0.200 | 0.035 | **0.690** | 0.136 | -0.202 |
| I Talk all the time.^1^ | 0.049 | **0.554** | 0.197 | -0.319 | 0.047 | **0.560** | 0.188 | -0.322 |
| I am restless and can’t sit still. | -0.073 | **0.712** | 0.023 | 0.057 | -0.071 | **0.740** | 0.022 | 0.059 |
| I’m too impatient to wait for my turn. | 0.117 | **0.543** | -0.041 | 0.037 | 0.116 | **0.569** | -0.041 | 0.039 |
| I act before thinking about. | 0.030 | **0.469** | -0.015 | -0.055 | 0.029 | **0.491** | -0.015 | -0.057 |
| I am unable to concentrate on anything. | -0.144 | **0.643** | -0.039 | 0.307 | -0.144 | **0.687** | -0.039 | 0.326 |
| I do not listen to directions.^1^ | 0.047 | **0.556** | -0.104 | 0.072 | 0.047 | **0.583** | -0.103 | 0.076 |
| I forget things. | -0.009 | **0.421** | -0.001 | 0.207 | -0.009 | **0.450** | -0.001 | 0.220 |
| I usually do not feel at ease when I meet people I do not know too well. | 0.206 | -0.033 | 0.003 | **0.705** | 0.199 | 0.034 | 0.003 | **0.720** |
| I’m scared by and nervous about new things and situation. | 0.132 | 0.016 | 0.087 | **0.722** | 0.128 | 0.017 | 0.084 | **0.744** |
| I am the kind of person who is excessively sensitive and easily hurt.^1^ | 0.126 | 0.104 | 0.061 | **0.470** | 0.125 | 0.110 | 0.061 | **0.496** |
| Even though I know I am right I often have great difficulty getting my points across | -0.005 | 0.127 | -0.046 | **0.452** | -0.005 | 0.135 | -0.046 | **0.479** |
| I help others when they need it. | -0.006 | 0.024 | **0.737** | -0.004 | -0.006 | 0.025 | **0.740** | -0.004 |
| I am friendly to others. | -0.183 | -0.023 | **0.628** | 0.078 | -0.182 | 0.024 | **0.628** | 0.082 |
| I’m a person everyone can trust. | -0.076 | -0.113 | **0.600** | 0.019 | -0.076 | 0.119 | **0.601** | 0.020 |
| I try to solve difficult problems reasonably and consider other people.^1^ | 0.006 | -0.297 | **0.572** | -0.023 | 0.006 | 0.314 | **0.570** | -0.025 |
| I’m a person everyone can trust. | 0.001 | 0.149 | **0.599** | -0.048 | 0.001 | 0.158 | **0.601** | -0.051 |
| I sort out things through discussion. | -0.169 | 0.037 | **0.497** | -0.063 | -0.168 | 0.040 | **0.497** | -0.067 |
| I always do my tasks. | 0.080 | -0.438 | **0.380** | 0.007 | 0.079 | 0.461 | **0.377** | 0.008 |
| I am calm and patient.^1^ | -0.017 | -0.527 | **0.313** | 0.126 | -0.017 | 0.545 | **0.306** | 0.129 |

Note.^1^ Thresholds cannot be set equal between boys and girls.

Table S19. Measurement invariance test results for hyperactive/inattentive, aggressive behavior, anxious behavior and prosocial factors between boys and girls evaluated by their co-twin at age 17.

| Model | $\chi^{2}$ | df | CFI | TLI | RMSEA | SRMR | $\Delta$ CFI | $\Delta$ RMSEA | $\Delta$ SRMR |
| --- | --- | --- | --- | --- | --- | --- | --- | --- | --- |
| M1 | 2701.563 | 455 | 0.961 | 0.945 | 0.048 | 0.028 | NA | NA | NA |
| M2 | 1821.098 | 543 | 0.978 | 0.974 | 0.033 | 0.031 | 0.017 | -0.015 | 0.003 |
| M3 | 2814.016 | 590 | 0.962 | 0.958 | 0.042 | 0.033 | -0.015 | 0.007 | 0.002 |
| M3^1^ | 2129.119 | 578 | 0.973 | 0.970 | 0.036 | 0.031 | -0.002 | 0.000 | 0.001 |
| M4 | 2734.927 | 616 | 0.963 | 0.961 | 0.040 | 0.033 | 0.001 | -0.002 | 0.000 |
| M4^1^ | 1988.758 | 604 | 0.976 | 0.974 | 0.033 | 0.032 | 0.013 | -0.003 | 0.001 |

Note. M2 model fits the data better than M1 model. ^1^ Partial invariance holds and thresholds for four items were freely estimated; corresponding residual variance is set to one.

Table S20. Factor correlations, means and variances for hyperactive/inattentive, aggressive behavior, anxious behavior and prosocial factors between boys and girls evaluated by their co-twin at age 17.

|  | Boys | | | | Girls | | | |
| --- | --- | --- | --- | --- | --- | --- | --- | --- |
|  | Aggressive behavior | Hyperactive/Inattentive | Prosocial | Anxious behavior | Aggressive behavior | Hyperactive/Inattentive | Prosocial | Anxious behavior |
| Aggressive behavior | 1.0 |  |  |  | 1.07 |  |  |  |
| Hyperactive/Inattentive | 0.61 | 1.0 |  |  | 0.56 | 1.03 |  |  |
| Prosocial | -0.31 | -0.29 | 1.0 |  | -0.36 | -0.30 | 0.94 |  |
| Anxious behavior | 0.07 | 0.05 | -0.17 | 1.0 | 0.02 | -0.09^3^ | -0.13 | 1.03 |
| Means | 0 | 0 | 0 | 0 | 0.30^3^ | -0.13^2^ | 0.49^3^ | 0.08^1^ |

Note. Difference between boys and girls ^1^ p < 0.05, ^2^ p < 0.01 and ^3^ p < 0.001.

Table S21. Standardized factor loadings for hyperactive/inattentive, aggressive behavior, anxious behavior and prosocial factors between boys and girls evaluated by their co-twin at age 17.

|  | Boys | | | | Girls | | | |
| --- | --- | --- | --- | --- | --- | --- | --- | --- |
|  | Aggressive behavior | Hyperactive/Inattentive | Prosocial | Anxious behavior | Aggressive behavior | Hyperactive/Inattentive | Prosocial | Anxious behavior |
| I often become angry, and I easily get involved in quarrels or fights | **0.625** | 0.104 | -0.163 | 0.029 | **0.637** | 0.104 | -0.156 | 0.029 |
| When people yell me, I yell back. | **0.616** | 0.040 | 0.044 | -0.204 | **0.626** | 0.040 | 0.041 | -0.203 |
| I sometimes feel the desire to tease, to annoy, or to attach another person without reason.^1^ | **0.515** | 0.125 | -0.104 | 0.056 | **0.529** | 0.125 | -0.100 | 0.057 |
| Given enough provocation, I may hit another person ^1^ | **0.750** | -0.107 | -0.114 | -0.008 | **0.757** | -0.105 | -0.108 | -0.008 |
| I spread rumors about other people’s personal matter when I am mad at them. | **0.467** | 0.105 | -0.191 | 0.109 | **0.481** | 0.106 | -0.183 | 0.110 |
| When I am mad at someone, I sometimes decide may exclude him/her. | **0.543** | 0.004 | -0.069 | 0.082 | **0.557** | 0.004 | -0.066 | 0.082 |
| I am hyperactive. | 0.165 | **0.628** | 0.153 | -0.144 | 0.168 | **0.627** | 0.146 | -0.144 |
| I Talk all the time. | 0.249 | **0.500** | 0.196 | -0.294 | 0.252 | **0.495** | 0.186 | -0.291 |
| I am restless and can’t sit still. | -0.047 | **0.805** | 0.043 | -0.018 | -0.048 | **0.807** | 0.041 | -0.018 |
| I’m too impatient to wait for my turn. | 0.101 | **0.660** | -0.091 | 0.039 | 0.104 | **0.668** | -0.088 | 0.040 |
| I act before thinking about. | 0.047 | **0.525** | -0.019 | -0.016 | 0.048 | **0.529** | -0.018 | -0.016 |
| I am unable to concentrate on anything. | -0.138 | **0.783** | -0.049 | 0.154 | -0.144 | **0.797** | -0.048 | 0.157 |
| I do not listen to directions. | 0.019 | **0.650** | -0.149 | 0.082 | 0.020 | **0.661** | -0.145 | 0.084 |
| I forget things. | -0.036 | **0.465** | -0.015 | 0.150 | -0.038 | **0.474** | -0.015 | 0.153 |
| I usually do not feel at ease when I meet people I do not know too well. | 0.010 | -0.073 | -0.012 | **0.799** | 0.011 | -0.073 | -0.012 | **0.797** |
| I’m scared by and nervous about new things and situation. | 0.124 | 0.013 | 0.111 | **0.800** | 0.128 | 0.013 | 0.107 | **0.807** |
| I am the kind of person who is excessively sensitive and easily hurt.^1^ | 0.197 | 0.061 | 0.068 | **0.451** | 0.205 | 0.062 | 0.066 | **0.459** |
| Even though I know I am right I often have great difficulty getting my points across | -0.080 | 0.189 | -0.017 | **0.403** | -0.083 | 0.193 | -0.017 | **0.411** |
| I help others when they need it. | 0.040 | -0.016 | **0.810** | -0.023 | 0.042 | -0.016 | **0.802** | -0.024 |
| I am friendly to others. | -0.214 | 0.024 | **0.722** | 0.079 | -0.223 | 0.024 | **0.702** | 0.081 |
| I’m a person everyone can trust. | -0.113 | -0.124 | **0.686** | 0.040 | -0.118 | -0.127 | **0.670** | 0.041 |
| I try to solve difficult problems reasonably and consider other people. | -0.021 | -0.274 | **0.630** | -0.036 | -0.022 | -0.282 | **0.618** | -0.037 |
| I’m a person everyone can trust. | 0.056 | 0.066 | **0.758** | -0.031 | 0.059 | 0.068 | **0.749** | -0.032 |
| I sort out things through discussion. | -0.064 | -0.104 | **0.546** | -0.086 | -0.067 | -0.106 | **0.534** | -0.088 |
| I always do my tasks. | 0.133 | -0.495 | **0.416** | 0.016 | 0.137 | -0.502 | **0.403** | 0.017 |
| I am calm and patient.^1^ | **0.625** | 0.104 | -0.163 | 0.029 | -0.067 | -0.452 | **0.363** | 0.157 |

Note. ^1^ Thresholds cannot be set equal between boys and girls.

Measurement invariance across informants was tested separately for girls and boys using confirmatory factor analysis, with only those items that theoretically belonged to a factor taken. Because weighting cannot be used to enriched data at age 14 in this part of invariance tests (i.e., weights are targeted to cases and therefore cannot be used when measures consist of other age measures. For example, in some cases a weight of 0.5 should be used at age 14 but should be 1.0 at other ages. However, using different weights for each age is not possible.), a sub-sample of enriched data was randomly chosen that corresponded to a proportion in the population. In all measurement invariance analyses, items were specified as categorical in the model, and the estimator used was the WLSMV in the Mplus statistical program (Muthén, L. K., & Muthén, B. O., 1998–2017). Because twins are more similar than two randomly selected individuals, the standard errors are corrected using the COMPLEX option in Mplus.

Each invariance test is based on hierarchically testing invariance levels with nested models M1–M4.

M1. Configural invariance – factor loadings and thresholds are freely estimated.

M2. Metric (weak) invariance – factor loadings are set equal, and thresholds are freely estimated.

M3. Scalar (strong) invariance – factor loadings and thresholds are set equal, but residual variances are freely estimated.

M4. Residual (strict) invariance – factor loadings, thresholds are set equal.

Partial scalar invariance held for the Hyperactive/Inattentive behavior in both girls’ and boys’ data and for Prosocial behavior in boys’ data across all ages and informants. For girls, partial scalar invariance held across age and informants, showing three invariance groups: 1) across teacher ratings at age 12 and 14, self and co-twin ratings at age 14 and co-twin ratings at age 17; 2) across parental ratings at age 12 and teacher, co-twin and self-ratings at age 14 and co-twin ratings at age 17; and 3) across self- and co-twin ratings at age 14 and 17. For Anxious behavior, the level of invariance achieved was metric for girls and boys across all ages and informants. For Aggressive behavior, partial scalar invariance held partly across informants: 1) across all informants at age 12 and 14 and 2) across self- and co-twin ratings at age 17.

Table S22. Measurement invariance test of aggressive behavior scale across informants for girls.

| Model | $\chi^{2}$ | df | CFI | TLI | RMSEA | SRMR | $\Delta$ CFI | $\Delta$ RMSEA | $\Delta$ SRMR |
| --- | --- | --- | --- | --- | --- | --- | --- | --- | --- |
| M1 | 1543.413 | 798 | 0.968 | 0.966 | 0.019 | 0.058 | NA | NA | NA |
| M2 | 1618.973 | 828 | 0.966 | 0.965 | 0.019 | 0.060 | -0.002 | 0.000 | 0.002 |
| M3 | 4094.770 | 894 | 0.863 | 0.868 | 0.037 | 0.069 | -0.142 | -0.019 | 0.006 |
| M3^1^ | 1737.912 | 863 | 0.963 | 0.963 | 0.020 | 0.059 | -0.008 | 0.002 | 0.000 |
| M4 | 5035.985 | 929 | 0.824 | 0.837 | 0.041 | 0.066 | 0.039 | 0.004 | -0.003 |
| M4^1^ | 1886.491 | 896 | 0.958 | 0.959 | 0.021 | 0.060 | -0.005 | 0.001 | 0.001 |

Note. Model set as M1 configural, M2 metric invariance, M3 scalar invariance and M4 strict invariance.

^1^ Some of the thresholds are freely estimated and corresponding residual variance is set to one (see Table S23).

Table S23. Threshold equality across informants in aggressive behavior scale for girls.

| Items | T12 | T14 | P12 | S14 | C14 | S17 | C17 |
| --- | --- | --- | --- | --- | --- | --- | --- |
| If he/she gets angry at someone, he/she might hit, push, kick, or throw something at hem/her.  I often become angry, and I easily get involved in quarrels or fights | 1 | 1 | 2 | 3 | 3 | 4 | 4 |
| Scolds people he/she is upset with.  When people yell me, I yell back. | 5 | 5 | 6 | 7 | 8 | 9 | 9 |
| Teases and attacks another without a reason.  I sometimes feel the desire to tease, to annoy, or to attach another person without reason. | 10 | 10 | 10 | 10 | 10 | 10 | 10 |
| Bullies smaller and weaker kids.  Given enough provocation, I may hit another person. | 11 | 11 | 11 | 11 | 11 | 11 | 11 |
| Spreads rumors about other people’s personal matters when he/she is mad at them.  I spread rumors about other people’s personal matters when I’m mad at them. | 12 | 12 | 12 | 12 | 12 | 13 | 14 |
| Excludes people from the group by saying, for example, “We don’t want to be with him/her”.  When I am mad at someone, I sometimes decide to exclude him/her | 15 | 16 | 16 | 16 | 16 | 17 | 17 |

Note. Number tells which of the thresholds are set as equal across informants. T12 Teacher evaluation at age 12. T14 Teacher evaluation at age 14. P12 Parent evaluation at age 12. S14 Self-evaluation at age 14. C14 Co-twin evaluation at age 14. S17 Self-evaluation at age 17. C17 Co-twin evaluation at age 17.

Table S24. Correlation between informants for aggressive behavior scale for girls.

|  | T12 | T14 | P12 | S14 | C14 | S17 | C17 |
| --- | --- | --- | --- | --- | --- | --- | --- |
| T12 | 1.0 |  |  |  |  |  |  |
| T14 | 0.37 | 1.0 |  |  |  |  |  |
| P12 | 0.27 | 0.21 | 1.0 |  |  |  |  |
| S14 | 0.21 | 0.10^1^ | 0.20^2^ | 1.0 |  |  |  |
| C14 | 0.26 | 0.32 | 0.37 | 0.42 | 1.0 |  |  |
| S17 | 0.27 | 0.23 | 0.29 | 0.54 | 0.37 | 1.0 |  |
| C17 | 0.34 | 0.25 | 0.32 | 0.40 | 0.62 | 0.67 | 1.0 |

Note. All correlations are statistically significant at p < 0.001 level, except ^1^ non-significant and ^2^ p < 0.02.

Table S25. Mean, variance and 95% confidence intervals (CI) of informants for aggressive behavior scale of girls.

|  | Mean | Lo 95% CI | Hi 95% CI | Var | Lo 95% CI | Hi 95% CI |
| --- | --- | --- | --- | --- | --- | --- |
| T12^1^ | 0 | NA | NA | 1 | NA | NA |
| T14^1^ | -0.64 | -0.77 | -0.51 | 1.16 | 0.93 | 1.39 |
| P12^1^ | 0.13 | 0.05 | 0.21 | 0.35 | 0.29 | 0.40 |
| S14^1^ | -0.03 | -0.13 | 0.07 | 0.34 | 0.27 | 0.42 |
| C14^1^ | 0.15 | 0.05 | 0.25 | 0.50 | 0.40 | 0.60 |
| S17^2^ | 1.23 | 1.15 | 1.31 | 0.22 | 0.19 | 0.25 |
| C17^2^ | 1.29 | 1.20 | 1.38 | 0.33 | 0.28 | 0.38 |

Note. ^1,2^ at least three items’ thresholds are set as equal and having equal superscript informants means they can be compared to each other.

All variances can be compared to each other.

Table S26. Measurement invariance tests of hyperactive/inattentive scale across informants for girls.

| Model | $\chi^{2}$ | df | CFI | TLI | RMSEA | SRMR | $\Delta\chi^{2}$ | $\Delta$ df | $\Delta p$-value | $\Delta$ CFI | $\Delta$ RMSEA | $\Delta$ SRMR |
| --- | --- | --- | --- | --- | --- | --- | --- | --- | --- | --- | --- | --- |
| M1 | 3876.362 | 1463 | 0.949 | 0.946 | 0.025 | 0.054 | NA | NA | NA | NA | NA | NA |
| M2 | 3874.323 | 1505 | 0.950 | 0.948 | 0.024 | 0.058 | 187.744 | 42 | < 0.001 | -0.001 | -0.001 | -0.004 |
| M3 | 5306.870 | 1595 | 0.921 | 0.924 | 0.030 | 0.057 | 352.643 | 48 | < 0.001 | -0.03 | -0.028 | 0.002 |
| M3^1^ | 4267.671 | 1568 | 0.943 | 0.944 | 0.026 | 0.055 | 214.071 | 45 | < 0.001 | -0.007 | 0.001 | 0 |
| M4 | 5424.445 | 1643 | 0.920 | 0.925 | 0.030 | 0.060 | 3132.115 | 138 | < 0.001 | 0.001 | 0 | 0.003 |
| M4^1^ | 4296.148 | 1613 | 0.943 | 0.946 | 0.025 | 0.058 | 802.233 | 108 | < 0.001 | 0 | -0.001 | 0.003 |

Note. Model set as M1 configural, M2 metric invariance, M3 scalar invariance and M4 strict invariance.

^1^ Some of the thresholds are freely estimated and the corresponding residual variance is set to one (see Table S27).

Table S27. Threshold equality across informants in hyperactive/inattentive scale for girls.

| Items | T12 | T14 | P12 | S14 | C14 | S17 | C17 |
| --- | --- | --- | --- | --- | --- | --- | --- |
| Is hyperactive.  I am hyperactive. | 1 | 1 | 1 | 1 | 1 | 1 | 1 |
| Talks all the time.  I talk all the time. | 2 | 2 | 2 | 2 | 2 | 2 | 2 |
| Is restless and cannot stay put during class.  I am restless and can’t sit still. | 3 | 3 | 3 | 3 | 3 | 4 | 3 |
| Is too impatient to wait for s/her turn.  I’m too impatient to wait for my turn. | 5 | 6 | 7 | 6 | 6 | 6 | 6 |
| Acts before thinking.  I act before thinking. | 8 | 8 | 8 | 8 | 8 | 8 | 8 |
| Is unable to concentrate on anything  I am unable to concentrate on anything. | 9 | 9 | 10 | 9 | 9 | 11 | 9 |
| Does not listen to directions.  I do not listen to directions. | 12 | 12 | 12 | 13 | 13 | 13 | 13 |
| Is forgetful.  I forget things | 14 | 14 | 14 | 15 | 15 | 16 | 17 |

Note. Number tells which of the thresholds are set as equal across informants. T12 Teacher evaluation at age 12. T14 Teacher evaluation at age 14. P12 Parent evaluation at age 12. S14 Self-evaluation at age 14. C14 Co-twin evaluation at age 14. S17 Self-evaluation at age 17. C17 Co-twin evaluation at age 17.

Table S28. Correlation between informants for hyperactive/inattentive scale for girls.

|  | T12 | T14 | P12 | S14 | C14 | S17 | C17 |
| --- | --- | --- | --- | --- | --- | --- | --- |
| T12 | 1.0 |  |  |  |  |  |  |
| T14 | 0.49 | 1.0 |  |  |  |  |  |
| P12 | 0.46 | 0.40 | 1.0 |  |  |  |  |
| S14 | 0.35 | 0.50 | 0.42 | 1.0 |  |  |  |
| C14 | 0.46 | 0.52 | 0.50 | 0.64 | 1.0 |  |  |
| S17 | 0.34 | 0.35 | 0.37 | 0.71 | 0.50 | 1.0 |  |
| C17 | 0.34 | 0.32 | 0.41 | 0.57 | 0.68 | 0.68 | 1.0 |

Note. All correlations are statistically significant at p < 0.001 level.

Table S29. Mean, variance and 95% confidence intervals (CI) of informants for hyperactive/inattentive scale of girls.

|  | Mean | Lo 95% CI | Hi 95% CI | Var | Lo 95% CI | Hi 95% CI |
| --- | --- | --- | --- | --- | --- | --- |
| T12 | 0 | NA | NA | 1 | NA | NA |
| T14 | -0.38 | -0.47 | -0.28 | 1.43 | 1.20 | 1.66 |
| P12 | 0.42 | 0.36 | 0.47 | 0.44 | 0.39 | 0.50 |
| S14 | 0.79 | 0.72 | 0.86 | 0.32 | 0.26 | 0.38 |
| C14 | 0.62 | 0.54 | 0.69 | 0.56 | 0.47 | 0.65 |
| S17 | 0.84 | 0.77 | 0.90 | 0.31 | 0.26 | 0.35 |
| C17 | 0.86 | 0.79 | 0.92 | 0.44 | 0.38 | 0.50 |

Note. All means and variances can be compared to each other.

Table S30. Measurement invariance tests of anxious behavior scale across informants for girls.

| Model | $\chi^{2}$ | df | CFI | TLI | RMSEA | SRMR | $\Delta$ CFI | $\Delta$ RMSEA | $\Delta$ SRMR |
| --- | --- | --- | --- | --- | --- | --- | --- | --- | --- |
| M1 | 987.811 | 331 | 0.945 | 0.937 | 0.027 | 0.051 | NA | NA | NA |
| M2 | 1081.231 | 347 | 0.939 | 0.933 | 0.028 | 0.055 | 0.006 | 0.001 | -0.004 |
| M3 | 2824.516 | 386 | 0.796 | 0.800 | 0.049 | 0.063 | -0.167 | -0.005 | 0.006 |
| M4 | 3135.29 | 410 | 0.772 | 0.790 | 0.050 | 0.061 | 0.024 | 0.001 | -0.002 |

Note. Model set as M1 configural, M2 metric invariance, M3 scalar invariance and M4 strict invariance. Thresholds cannot be set as equal. The accepted model is M2.

Table S31. Correlation between informants for anxious behavior scale for girls.

|  | T12 | T14 | P12 | S14 | C14 | S17 | C17 |
| --- | --- | --- | --- | --- | --- | --- | --- |
| T12 | 1.0 |  |  |  |  |  |  |
| T14 | 0.45 | 1.0 |  |  |  |  |  |
| P12 | 0.41 | 0.36 | 1.0 |  |  |  |  |
| S14 | 0.19^1^ | 0.31 | 0.36 | 1.0 |  |  |  |
| C14 | 0.14^1^ | 0.25 | 0.34 | 0.58 | 1.0 |  |  |
| S17 | 0.23 | 0.25 | 0.30 | 0.65 | 0.39 | 1.0 |  |
| C17 | 0.26 | 0.22 | 0.37 | 0.39 | 0.61 | 0.61 | 1.0 |

Note. All correlations are statistically significant at p < 0.001 level, except ^1^ p < 0.01.

Table S32. Variance and 95% confidence intervals (CI) of informants for anxious behavior scale of girls.

|  | Var | Lo 95% CI | Hi 95% CI |
| --- | --- | --- | --- |
| T12 | 1 | NA | NA |
| T14 | 1.60 | 1.31 | 1.89 |
| P12 | 0.91 | 0.76 | 1.07 |
| S14 | 0.54 | 0.39 | 0.69 |
| C14 | 0.65 | 0.48 | 0.83 |
| S17 | 1.16 | 1.00 | 1.32 |
| C17 | 1.13 | 0.99 | 1.28 |

Note. All variances can be compared to each other.

Table S33. Measurement invariance tests of prosocial behavior scale across informants for girls.

| Model | $\chi^{2}$ | df | CFI | TLI | RMSEA | SRMR | $\Delta$ CFI | $\Delta$ RMSEA | $\Delta$ SRMR |
| --- | --- | --- | --- | --- | --- | --- | --- | --- | --- |
| M1 | 4903.023 | 1461 | 0.912 | 0.908 | 0.030 | 0.064 | NA | NA | NA |
| M2 | 4757.614 | 1496 | 0.917 | 0.914 | 0.029 | 0.065 | -0.005 | -0.001 | -0.001 |
| M3 | 8390.322 | 1584 | 0.827 | 0.831 | 0.040 | 0.069 | -0.088 | -0.025 | 0.004 |
| M3^1^ | 5261.162 | 1556 | 0.906 | 0.907 | 0.030 | 0.065 | -0.008 | 0 | 0.001 |
| M4 | 8350.430 | 1632 | 0.829 | 0.838 | 0.040 | 0.069 | -0.002 | 0 | 0 |
| M4^1^ | 5170.838 | 1589 | 0.909 | 0.912 | 0.029 | 0.066 | 0.003 | -0.001 | 0.001 |

Note. Model set as M1 configural, M2 metric invariance, M3 scalar invariance and M4 strict invariance.

^1^ Some of the thresholds are freely estimated and the corresponding residual variance is set to one (see Table S34).

Table S34. Threshold equality across informants in prosocial behavior scale for girls.

| Items from self and co-twin at age 17 | T12 | T14 | P12 | S14 | C14 | S17 | C17 |
| --- | --- | --- | --- | --- | --- | --- | --- |
| Helps others when they need it / I help others when they need it. | 1 | 1 | 1 | 1 | 1 | 2 | 1 |
| Is friendly to others / I am friendly to others. | 3 | 3 | 3 | 3 | 3 | 4 | 3 |
| Is a student everyone can trust / I’m a person everyone can trust. | 5 | 6 | 6 | 6 | 6 | 6 | 6 |
| Tries to act reasonably even in difficult situations /I try to solve difficult problems reasonably and consider other people. | 7 | 7 | 8 | 9 | 8 | 9 | 8 |
| Defends those weaker and smaller / I defend those who are weaker. | 10 | 10 | 11 | 12 | 12 | 12 | 12 |
| Sorts things out through discussion / I sort out things through discussion. | 13 | 13 | 14 | 13 | 13 | 13 | 13 |
| Is conscientious with homework / I always do my tasks. | 15 | 15 | 16 | 17 | 17 | 18 | 17 |
| Is patient and calm / I am calm and patient. | 19 | 19 | 20 | 20 | 20 | 21 | 21 |

Note. Number refers to the estimated parameter and tells if thresholds are set as equal across informants. T12 is teacher evaluation at age 12, T14 is teacher evaluation at age 14, P12 is parent evaluation at age 12, S14 is self-evaluation at age 14, C14 is co-twin evaluation at age 14, S17 is self-evaluation at age 17 and C17 is co-twin evaluation at age 17.

Table S35. Correlation between informants for prosocial behavior scale for girls.

|  | T12 | T14 | P12 | S14 | C14 | S17 | C17 |
| --- | --- | --- | --- | --- | --- | --- | --- |
| T12 | 1.0 |  |  |  |  |  |  |
| T14 | 0.46 | 1.0 |  |  |  |  |  |
| P12 | 0.37 | 0.22 | 1.0 |  |  |  |  |
| S14 | 0.22 | 0.38 | 0.33 | 1.0 |  |  |  |
| C14 | 0.27 | 0.47 | 0.44 | 0.54 | 1.0 |  |  |
| S17 | 0.15 | 0.19 | 0.30 | 0.65 | 0.40 | 1.0 |  |
| C17 | 0.24 | 0.26 | 0.32 | 0.39 | 0.64 | 0.47 | 1.0 |

Note. All correlations are statistically significant at p < 0.001.

Table S36. Mean, variance and 95% confidence intervals (CI) of informants for prosocial behavior scale of girls.

|  | Mean | Lo 95% CI | Hi 95% CI | Var | Lo 95% CI | Hi 95% CI |
| --- | --- | --- | --- | --- | --- | --- |
| T12^1^ | 0 | NA | NA | 1 | NA | NA |
| T14^1^ | 0.10 | 0.03 | 0.18 | 1.17 | 1.03 | 1.32 |
| P12^2^ | 0.41 | 0.34 | 0.48 | 0.67 | 0.59 | 075 |
| S14^1,2,3^ | -0.06 | -0.15 | 0.03 | 0.59 | 0.50 | 0.69 |
| C14^1,2,3^ | -0.08 | -0.17 | 0.02 | 0.88 | 0.75 | 1.01 |
| S17^3^ | 0.04 | -0.04 | 0.11 | 0.52 | 0.45 | 0.58 |
| C17^1,2,3^ | -0.07 | -0.14 | 0.00 | 0.82 | 0.73 | 0.92 |

Note. ^1,2,3^ at least three items’ thresholds are set as equal and having equal superscript informants means they can be compared.

All variances can be compared to each other.

Table S37. Measurement invariance test of aggressive behavior scale across informants for boys.

| Model |  | $\chi^{2}$ | df | CFI | TLI | RMSEA | SRMR | $\Delta$ CFI | $\Delta$ RMSEA | $\Delta$ SRMR |
| --- | --- | --- | --- | --- | --- | --- | --- | --- | --- | --- |
| M1 |  | 1681.719 | 798 | 0.974 | 0.972 | 0.020 | 0.050 | NA | NA | NA |
| M2 |  | 1887.458 | 828 | 0.969 | 0.968 | 0.022 | 0.055 | 0.005 | 0.002 | -0.005 |
| M3 |  | 5563.912 | 895 | 0.863 | 0.868 | 0.044 | 0.063 | -0.13 | -0.008 | 0.008 |
| M3^1^ |  | 2110.426 | 877 | 0.964 | 0.965 | 0.023 | 0.053 | -0.007 | 0.001 | 0 |
| M4 |  | 6440.045 | 930 | 0.839 | 0.851 | 0.047 | 0.063 | 0.024 | 0.003 | 0 |
| M4^1^ |  | 2218.371 | 906 | 0.962 | 0.963 | 0.023 | 0.055 | -0.002 | 0 | 0.002 |

Note. Model set as M1 configural, M2 metric invariance, M3 scalar invariance and M4 strict invariance.

^1^ Some of the thresholds are freely estimated and the corresponding residual variance is set to one (see Table S38).

Table S38. Threshold equality across informants in aggressive behavior scale for boys.

| Items from self and co-twin at age 17 | T12 | T14 | P12 | S14 | C14 | S17 | C17 |
| --- | --- | --- | --- | --- | --- | --- | --- |
| If he/she gets angry at someone, he/she might hit, push, kick, or throw something at hem/her.  I often become angry, and I easily get involved in quarrels or fights | 1 | 1 | 1 | 1 | 1 | 2 | 2 |
| Scolds people he/she is upset with.  When people yell me, I yell back. | 3 | 3 | 3 | 3 | 3 | 4 | 4 |
| Teases and attacks another without a reason.  I sometimes feel the desire to tease, to annoy, or to attach another person without reason. | 5 | 6 | 7 | 7 | 7 | 7 | 7 |
| Bullies smaller and weaker kids.  Given enough provocation, I may hit another person. | 8 | 8 | 9 | 8 | 8 | 8 | 8 |
| Spreads rumors about other people’s personal matters when he/she is mad at them.  I spread rumors about other people’s personal matters when I’m mad at them. | 10 | 10 | 10 | 10 | 10 | 11 | 11 |
| Excludes people from the group by saying, for example, “We don’t want to be with him/her”.  When I am mad at someone, I sometimes decide to exclude him/her | 12 | 12 | 12 | 12 | 12 | 13 | 13 |

Note. Number tells which of the thresholds are set as equal across informants. T12 Teacher evaluation at age 12. T14 Teacher evaluation at age 14. P12 Parent evaluation at age 12. S14 Self-evaluation at age 14. C14 Co-twin evaluation at age 14. S17 Self-evaluation at age 17. C17 Co-twin evaluation at age 17.

Table S39. Correlation between informants for aggressive behavior scale for boys.

|  | T12 | T14 | P12 | S14 | C14 | S17 | C17 |
| --- | --- | --- | --- | --- | --- | --- | --- |
| T12 | 1.0 |  |  |  |  |  |  |
| T14 | 0.46 | 1.0 |  |  |  |  |  |
| P12 | 0.38 | 0.28 | 1.0 |  |  |  |  |
| S14 | 0.18 | 0.23 | 0.25 | 1.0 |  |  |  |
| C14 | 0.24 | 0.26 | 0.34 | 0.38 | 1.0 |  |  |
| S17 | 0.28 | 0.35 | 0.21 | 0.54 | 0.26 | 1.0 |  |
| C17 | 0.31 | 0.31 | 0.29 | 0.32 | 0.56 | 0.56 | 1.0 |

Note. All correlations are statistically significant at p < 0.001.

Table S40. Mean, variance and 95% confidence intervals (CI) of informants for aggressive behavior scale of boys.

|  | Mean | Lo 95% CI | Hi 95% CI | Var | Lo 95% CI | Hi 95% CI |
| --- | --- | --- | --- | --- | --- | --- |
| T12^1^ | 0 | NA | NA | 1 | NA | NA |
| T14^1^ | -0.65 | -0.74 | -0.55 | 1.05 | 0.88 | 1.23 |
| P12^1^ | 0.15 | 0.09 | 0.20 | 0.32 | 0.28 | 0.37 |
| S14^1^ | -0.05 | -0.12 | 0.03 | 0.27 | 0.22 | 0.32 |
| C14^1^ | 0.09 | 0.02 | 0.16 | 0.44 | 0.37 | 0.52 |
| S17^2^ | 0.80 | 0.73 | 0.86 | 0.19 | 0.17 | 0.22 |
| C17^2^ | 0.93 | 0.86 | 1.00 | 0.30 | 0.26 | 0.34 |

Note. ^1,2^ at least three items’ thresholds can be set as equal and having equal superscript informants means they can be compared.

All variances can be compared to each other.

Table S41. Measurement invariance tests of hyperactive/inattentive scale across informants for boys.

| Model | $\chi^{2}$ | df | CFI | TLI | RMSEA | SRMR | $\Delta$ CFI | $\Delta$ RMSEA | $\Delta$ SRMR |
| --- | --- | --- | --- | --- | --- | --- | --- | --- | --- |
| M1 | 4218.611 | 1463 | 0.960 | 0.958 | 0.027 | 0.055 | NA | NA | NA |
| M2 | 4440.587 | 1505 | 0.957 | 0.956 | 0.027 | 0.061 | 0.003 | 0 | -0.006 |
| M3 | 5569.425 | 1595 | 0.942 | 0.944 | 0.030 | 0.058 | -0.016 | -0.031 | 0.002 |
| M3^1^ | 4915.597 | 1575 | 0.951 | 0.952 | 0.028 | 0.057 | -0.007 | 0.001 | 0.001 |
| M4 | 5706.825 | 1643 | 0.941 | 0.944 | 0.030 | 0.063 | 0.001 | 0 | 0.005 |
| M4^1^ | 5033.170 | 1619 | 0.950 | 0.953 | 0.028 | 0.062 | -0.001 | 0 | 0.005 |

Note. Model set as M1 configural, M2 metric invariance, M3 scalar invariance and M4 strict invariance.

^1^ Some of the thresholds are freely estimated and the corresponding residual variance is set to one (see Table S42).

Table S42. Threshold equality across informants in hyperactive/inattentive scale for boys.

| Items from self and co-twin at age 17 | T12 | T14 | P12 | S14 | C14 | S17 | C17 |
| --- | --- | --- | --- | --- | --- | --- | --- |
| Is hyperactive / I am hyperactive. | 1 | 2 | 3 | 4 | 2 | 2 | 2 |
| Talks all the time / I talk all the time. | 5 | 5 | 5 | 6 | 5 | 5 | 5 |
| Is restless and cannot stay put during class / I am restless and can’t sit still. | 7 | 7 | 7 | 7 | 7 | 7 | 7 |
| Is too impatient to wait for s/her turn /I’m too impatient to wait for my turn. | 8 | 8 | 8 | 8 | 8 | 8 | 8 |
| Acts before thinking / I act before thinking. | 9 | 9 | 9 | 9 | 9 | 9 | 9 |
| Is unable to concentrate on anything / I am unable to concentrate on anything. | 10 | 11 | 12 | 11 | 11 | 11 | 11 |
| Does not listen to directions / I do not listen to directions. | 13 | 13 | 13 | 13 | 13 | 13 | 13 |
| Is forgetful / I forget things | 14 | 15 | 15 | 15 | 15 | 15 | 15 |

Note. Number tells which of the thresholds are set as equal across informants. T12 Teacher evaluation at age 12. T14 Teacher evaluation at age 14. P12 Parent evaluation at age 12. S14 Self-evaluation at age 14. C14 Co-twin evaluation at age 14. S17 Self-evaluation at age 17. C17 Co-twin evaluation at age 17.

Table S43. Correlation between informants for hyperactive/inattentive scale for boys.

|  | T12 | T14 | P12 | S14 | C14 | S17 | C17 |
| --- | --- | --- | --- | --- | --- | --- | --- |
| T12 | 1.0 |  |  |  |  |  |  |
| T14 | 0.54 | 1.0 |  |  |  |  |  |
| P12 | 0.52 | 0.40 | 1.0 |  |  |  |  |
| S14 | 0.41 | 0.53 | 0.36 | 1.0 |  |  |  |
| C14 | 0.46 | 0.51 | 0.38 | 0.54 | 1.0 |  |  |
| S17 | 0.36 | 0.45 | 0.37 | 0.71 | 0.46 | 1.0 |  |
| C17 | 0.40 | 0.42 | 0.43 | 0.48 | 0.68 | 0.59 | 1.0 |

Note. All correlations are statistically significant at p < 0.001.

Table S44. Mean, variance and 95% confidence intervals (CI) of informants for hyperactive/inattentive scale of boys.

|  | Mean | Lo 95% CI | Hi 95% CI | Var | Lo 95% CI | Hi 95% CI |
| --- | --- | --- | --- | --- | --- | --- |
| T12 | 0 | NA | NA | 1 | NA | NA |
| T14 | -0.43 | -0.51 | -0.35 | 1.23 | 1.07 | 1.39 |
| P12 | -0.11 | -0.16 | -0.06 | 0.38 | 0.34 | 0.42 |
| S14 | -0.21 | -0.28 | -0.15 | 0.25 | 0.20 | 0.29 |
| C14 | -0.06 | -0.12 | 0.01 | 0.42 | 0.36 | 0.48 |
| S17 | -0.04 | -0.09 | 0.02 | 0.21 | 0.18 | 0.24 |
| C17 | -0.03 | -0.09 | 0.02 | 0.34 | 0.30 | 0.38 |

Note. All means and variances can be compared to each other.

Table S45. Measurement invariance tests of anxious behavior scale across informants for boys.

| Model | $\chi^{2}$ | df | CFI | TLI | RMSEA | SRMR | $\Delta$ CFI | $\Delta$ RMSEA | $\Delta$ SRMR |
| --- | --- | --- | --- | --- | --- | --- | --- | --- | --- |
| M1 | 769.588 | 331 | 0.953 | 0.947 | 0.022 | 0.046 | NA | NA | NA |
| M2 | 876.834 | 347 | 0.944 | 0.939 | 0.024 | 0.050 | 0.009 | 0.002 | -0.004 |
| M3 | 3638.005 | 386 | 0.655 | 0.662 | 0.056 | 0.068 | -0.382 | 0.011 | 0.014 |
| M4 | 4533.233 | 410 | 0.562 | 0.596 | 0.061 | 0.064 | 0.093 | 0.005 | -0.004 |

Note. Model set as M1 configural, M2 metric invariance, M3 scalar invariance and M4 strict invariance. Thresholds cannot be set as equal. The accepted model is M2.

Table S46. Correlation between informants for anxious behavior scale for boys.

|  | T12 | T14 | P12 | S14 | C14 | S17 | C17 |
| --- | --- | --- | --- | --- | --- | --- | --- |
| T12 | 1.0 |  |  |  |  |  |  |
| T14 | 0.34 | 1.0 |  |  |  |  |  |
| P12 | 0.33 | 0.24 | 1.0 |  |  |  |  |
| S14 | 0.11^1^ | 0.34 | 0.37 | 1.0 |  |  |  |
| C14 | 0.15^2^ | 0.26 | 0.34 | 0.42 | 1.0 |  |  |
| S17 | 0.16 | 0.23 | 0.28 | 0.46 | 0.25 | 1.0 |  |
| C17 | 0.15 | 0.20 | 0.30 | 0.30 | 0.53 | 0.44 | 1.0 |

Note. All correlations are statistically significant at p < 0.001 level, except ^1^ non-significant and ^2^ p < 0.01.

Table S47. Variance and 95% confidence intervals (CI) of informants for anxious behavior scale of boys.

|  | Var | Lo 95% CI | Hi 95% CI |
| --- | --- | --- | --- |
| T12 | 1 | NA | NA |
| T14 | 1.52 | 1.25 | 1.80 |
| P12 | 0.83 | 0.70 | 0.97 |
| S14 | 0.65 | 0.48 | 0.83 |
| C14 | 0.84 | 0.62 | 1.05 |
| S17 | 0.92 | 0.79 | 1.06 |
| C17 | 1.09 | 0.94 | 1.23 |

Note. All variances can be compared to each other.

Table S48. Measurement invariance tests of prosocial behavior scale across informants for boys.

| Model | $\chi^{2}$ | df | CFI | TLI | RMSEA | SRMR | $\Delta$ CFI | $\Delta$ RMSEA | $\Delta$ SRMR |
| --- | --- | --- | --- | --- | --- | --- | --- | --- | --- |
| M1 | 5495.460 | 1470 | 0.916 | 0.912 | 0.032 | 0.063 | NA | NA | NA |
| M2 | 5383.950 | 1505 | 0.919 | 0.917 | 0.031 | 0.065 | -0.003 | -0.001 | -0.002 |
| M3 | 7336.847 | 1595 | 0.880 | 0.884 | 0.037 | 0.064 | -0.036 | -0.029 | 0.002 |
| M3^1^ | 5874.045 | 1565 | 0.910 | 0.912 | 0.032 | 0.063 | -0.008 | 0 | 0 |
| M4 | 7250,693 | 1643 | 0.883 | 0.890 | 0.036 | 0.067 | -0.003 | -0.001 | 0.003 |
| M4^1^ | 5857.600 | 1607 | 0.911 | 0.915 | 0.031 | 0.065 | 0.001 | -0.001 | 0.002 |

Note. Model set as M1 configural, M2 metric invariance, M3 scalar invariance and M4 strict invariance.

^1^ Some of the thresholds are freely estimated and the corresponding residual variance is set to one (see Table S49).

Table S49. Threshold equality across informants in prosocial behavior scale for boys.

| Items from self and co-twin at age 17 | T12 | T14 | P12 | S14 | C14 | S17 | C17 |
| --- | --- | --- | --- | --- | --- | --- | --- |
| Helps others when they need it / I help others when they need it. | 1 | 2 | 1 | 1 | 1 | 1 | 1 |
| Is friendly to others / I am friendly to others. | 3 | 3 | 3 | 3 | 3 | 3 | 3 |
| Is a student everyone can trust / I’m a person everyone can trust. | 4 | 4 | 4 | 4 | 4 | 4 | 5 |
| Tries to act reasonably even in difficult situations / I try to solve difficult problems reasonably and consider other people. | 6 | 7 | 7 | 8 | 9 | 7 | 7 |
| Defends those weaker and smaller / I defend those who are weaker. | 10 | 11 | 12 | 13 | 13 | 13 | 13 |
| Sorts things out through discussion / I sort out things through discussion. | 14 | 14 | 14 | 14 | 14 | 14 | 14 |
| Is conscientious with homework / I always do my tasks. | 15 | 15 | 15 | 15 | 15 | 16 | 15 |
| Is patient and calm. / I am calm and patient. | 17 | 17 | 18 | 17 | 17 | 17 | 17 |

Note. Number refers to the estimated parameter and tells if thresholds are set as equal across informants. T12 is teacher evaluation at age 12, T14 is teacher evaluation at age 14, P12 is parent evaluation at age 12, S14 is self-evaluation at age 14, C14 is co-twin evaluation at age 14, S17 is self-evaluation at age 17 and C17 is co-twin evaluation at age 17.

Table S50. Correlation between informants for prosocial behavior scale for boys.

|  |  | T12 | T14 | P12 | S14 | C14 | S17 | C17 |
| --- | --- | --- | --- | --- | --- | --- | --- | --- |
| T12 |  | 1.0 |  |  |  |  |  |  |
| T14 |  | 0.49 | 1.0 |  |  |  |  |  |
| P12 |  | 0.40 | 0.29 | 1.0 |  |  |  |  |
| S14 |  | 0.30 | 0.36 | 0.20 | 1.0 |  |  |  |
| C14 |  | 0.38 | 0.39 | 0.32 | 0.45 | 1.0 |  |  |
| S17 |  | 0.28 | 0.26 | 0.30 | 0.60 | 0.32 | 1.0 |  |
| C17 |  | 0.31 | 0.28 | 0.37 | 0.36 | 0.63 | 0.44 | 1.0 |

Note. All correlations are statistically significant at p < 0.001 level.

Table S51. Mean, variance and 95% confidence intervals (CI) of informants for prosocial behavior scale of boys.

|  | Mean | Lo 95% CI | Hi 95% CI | Var | Lo 95% CI | Hi 95% CI |
| --- | --- | --- | --- | --- | --- | --- |
| T12 | 0 | NA | NA | 1 | NA | NA |
| T14 | 0.33 | 0.26 | 0.40 | 1.08 | 0.96 | 1.21 |
| P12 | 0.58 | 0.52 | 0.64 | 0.52 | 0.46 | 0.58 |
| S14 | 0.15 | 0.08 | 0.22 | 0.43 | 0.36 | 0.50 |
| C14 | -0.18 | -0.25 | -0.10 | 0.61 | 0.52 | 0.71 |
| S17 | 0.34 | 0.28 | 0.40 | 0.38 | 0.33 | 0.42 |
| C17 | -0.02 | -0.09 | 0.04 | -0.02 | -0.10 | 0.05 |

Note. All means and variances can be compared to each other.

**References**

Chen, F. F. (2007). Sensitivity of goodness of fit indexes to lack of measurement invariance. Structural Equation Modeling: A Multidisciplinary Journal, 14(3), 464e504. <https://doi.org/10.1080/10705510701301834>

Hu, L., & Bentler, P. M. (1999). Cutoff criteria for fit indexes in covariance structure analysis: Conventional criteria versus new alternatives. Structural Equation Modeling: A Multidisciplinary Journal, 6(1), 1–55. <https://doi.org/10.1080/10705519909540118>

Muthén, L. K., & Muthén, B. O. (1998–2017). Mplus. Statistical analysis with latent variables. User’s guide (8th ed.). Los Angeles, CA: Muthén and Muthén.

Putnick, D. L., & Bornstein, M. H. (2016). Measurement invariance conventions and reporting: The state of the art and future directions for psychological research. Developmental Review, 41, 71–90. <https://doi.org/10.1016/j.dr.2016.06.004>
