# Supplementary material for: Self-regulation of socioemotional behavior in twin adolescents: Structural validation of a multidimensional inventory
Source: PLOS Ment Health. 2025 Oct 9;2(10):e0000448. doi: 10.1371/journal.pmen.0000448 (PMC12798259; doi:10.1371/journal.pmen.0000448)
Supplement: Supplementary file 6 [file pmen.0000448.s003.docx]

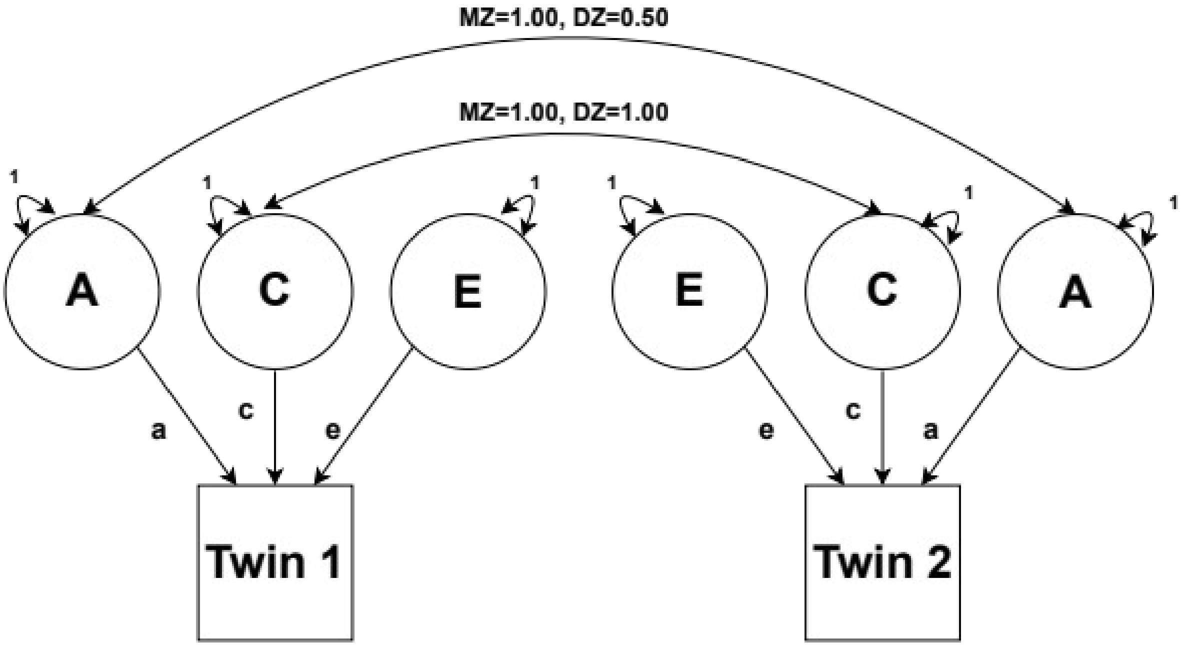

MZ=1.00, DZ=0.50
MZ=1.00, DZ=1.00
1
1
1
1
1
1
A
C
E
E
C
A
a
c
e
e
c
a
Twin 1
Twin 2
