# Supplementary material for: Self-regulation of socioemotional behavior in twin adolescents: Structural validation of a multidimensional inventory
Source: PLOS Ment Health. 2025 Oct 9;2(10):e0000448. doi: 10.1371/journal.pmen.0000448 (PMC12798259; doi:10.1371/journal.pmen.0000448)
Supplement: Attachment — Submitted filename: Peer_review_rebuttal_report_02092025.docx [file pmen.0000448.s007.docx]

Peer Review Response to Reviewers

Revision deadline (extended): Sept 7, 2025

PMEN-D-25-00146
Self-regulation of socioemotional behavior in twin adolescents: structural validation of a multidimensional inventory
PLOS Mental Health

Dear Dr. Whipp,

Thank you for submitting your manuscript to PLOS Mental Health. After careful consideration, we feel that it has merit but does not fully meet PLOS Mental Health’s publication criteria as it currently stands. Therefore, we invite you to submit a revised version of the manuscript that addresses the points raised during the review process.

As you can see, the Reviewer 2 suggests some changes to your manuscript. After considering the review and reading the paper myself, I offer a number of points to address in a potential revision, as well.

   1. Please clarify the use of term “bi-factor”. Bifactor models, which are ubiquitous in the structural modeling of psychopathology and regulatory capacities, include superordinate general factors as well as subordinate specific factors. It is not clear which model is used in your study.

References

Clark, D. A., Hicks, B. M., Angstadt, M., Rutherford, S., Taxali, A., Hyde, L., ... & Sripada, C. (2021). The general factor of psychopathology in the adolescent brain cognitive development (ABCD) study: A comparison of alternative modeling approaches. Clinical Psychological Science, 9(2), 169-182.

Markon, K. E. (2019). Bifactor and hierarchical models: Specification, inference, and interpretation. Annual review of clinical psychology, 15(1), 51-69.

Snyder, H. R., Friedman, N. P., & Hankin, B. L. (2021). Associations between task performance and self-report measures of cognitive control: Shared versus distinct abilities. Assessment, 28(4), 1080-1096.

**Author response:**   Thank you for this important clarification question. We regret that the concept of a bifactor was used in the previous manuscript version by error. The factor for low versus high self-regulation was not meant to be a hierarchical “bifactor” (Markon, 2019) but rather a “bipolar factor” with opposite poles, as depicted by the two-dimensional impulse control model, as shown in Figure 1, now added to the manuscript. The bifactor model was also tested just to be quite sure, but it did not fit the data. This conceptual error has now been corrected in the manuscript (Abstract and page 6). The factors were also confirmed by a structural analysis (see page 10-11 and 15-18 for these Methods and Results of analyses, respectively) for two bipolar factors and the components of low and high self-regulation.

2. Please present the figure depiciting the MPNI structural model described in the first aim.

**Author response:** Good point. The description of the factor structure of the MPNI Form SERI is now presented in the Results section based on two- and four-exploratory factors models with fit indices. The two factors model is close to a good fit, but the four factors model fits the data better. In the four factors model, low self-regulation is divided into three components (Hyperactive/Inattentive behavior, Aggression, and Anxiety) and high self-regulation is depicted by Prosocial behavior. These factors correspond to the factors obtained by tentative analyses with the whole original MPNI (now presented in the Methods section for Analysis). Additionally, we have now included Figure 1 to depict the structural model.

  3. Please present a rationale for separating low and high self-regulation factors.

**Author response:**   There are two poles, low and high self-regulation, not two separate factors. See Figure 1 added to the manuscript (p. 7). The amount of self-regulation varies in quantity, as social activity.

   4. I specifically encourage you to consider your statistical approach. The hypothesised structure of the MPNI self-ratings at age 17 is presented at the end of the Introduction, therefore, I would expect to see it tested by structural equation modelling procedures. Even more so that you test measurement invariance later.

**Author response:**   Please, see response to question #2 above.

   5. The Analysis subsection describes measurement invariance testing. It might be helpful to present the findings in the main manuscript and include assessment of model fit for the total sample.

**Author response:**   The results of the invariance analyses have been added to the manuscript (p. 15-18). Tables are presented as Supplementary material (Supplementary Analyses 1).

Please submit your revised manuscript by Jul 22 2025 11:59PM. If you will need more time than this to complete your revisions, please reply to this message or contact the journal office at mentalhealth@plos.org. When you're ready to submit your revision, log on to https://www.editorialmanager.com/pmen/ and select the 'Submissions Needing Revision' folder to locate your manuscript file.

Please include the following items when submitting your revised manuscript:

- A rebuttal letter that responds to each point raised by the editor and reviewer(s). You should upload this letter as a separate file labeled 'Response to Reviewers'.
- A marked-up copy of your manuscript that highlights changes made to the original version. You should upload this as a separate file labeled 'Revised Manuscript with Track Changes'.
- An unmarked version of your revised paper without tracked changes. You should upload this as a separate file labeled 'Manuscript'.

Guidelines for resubmitting your figure files are available below the reviewer comments at the end of this letter.

We look forward to receiving your revised manuscript.

Kind regards,

Helena R. Slobodskaya, M.D., Ph.D., D.Sc.
Academic Editor
PLOS Mental Health

Journal Requirements:
1. We have noticed that you have uploaded Supporting Information files, but you have not included a list of legends. Please add a full list of legends for your Supporting Information files after the references list.

**Author response:**   A separate file entitled “List of Legends for Figures and Tables” has now been created as a separate file (it was also placed at the end of the manuscript file after the reference list. (Additionally, the Figures and Tables also had their titles/legends listed in the main body of the manuscript, so this was not changed)

2. Please review your reference list to ensure that it is complete and correct. If you have cited papers that have been retracted, please include the rationale for doing so in the manuscript text, or remove these references and replace them with relevant current references. Any changes to the reference list should be mentioned in the rebuttal letter that accompanies your revised manuscript. If you need to cite a retracted article, indicate the article’s retracted status in the References list and also include a citation and full reference for the retraction notice.

**Author response:**   The references have all been checked: all references mentioned in the main text appear in the reference list, and there are no references in the list that are unused. Three more references were added to a paragraph in the Discussion because we had relevant references using our own FinnTwin12 data to support the point that we were making and think this makes the point/paper stronger. We can also remove them if the editor desires. We also added Goodman et al. (2010) as a reference to clarify a point in the Discussion (see also response to Reviewer #1 Limitations/Critique item #9). Lastly, two references were added to support the structural equation modeling requested in this peer review revision (Hu et al. 2009 and Putnick & Bornstein 2016).

Additional Editor Comments (if provided):


[Note: HTML markup is below. Please do not edit.]

Reviewers' comments:

Reviewer's Responses to Questions

**Comments to the Author**

1. Does this manuscript meet PLOS Mental Health’s [publication criteria](https://track.editorialmanager.com/CL0/https:%2F%2Fjournals.plos.org%2Fmentalhealth%2Fs%2Fcriteria-for-publication/1/010f01979a9ebd86-4edd0b63-6ee8-4a46-b8f4-aaa74b1ea0f0-000000/ZmzFo_fCkxjpymUoC9wV60f77cRN0ClQigaDOjhjVnU=216)? Is the manuscript technically sound, and do the data support the conclusions? The manuscript must describe methodologically and ethically rigorous research with conclusions that are appropriately drawn based on the data presented.

Reviewer #1: Yes

2. Has the statistical analysis been performed appropriately and rigorously?

Reviewer #1: Yes

3. Have the authors made all data underlying the findings in their manuscript fully available (please refer to the Data Availability Statement at the start of the manuscript PDF file)?

The [PLOS Data policy](https://track.editorialmanager.com/CL0/https:%2F%2Fjournals.plos.org%2Fmentalhealth%2Fs%2Fdata-availability/1/010f01979a9ebd86-4edd0b63-6ee8-4a46-b8f4-aaa74b1ea0f0-000000/JDL3y1Lwg6hyS7ZkEcln87hwuHUaW9jkssq0O0DunSw=216) requires authors to make all data underlying the findings described in their manuscript fully available without restriction, with rare exception. The data should be provided as part of the manuscript or its supporting information, or deposited to a public repository. For example, in addition to summary statistics, the data points behind means, medians and variance measures should be available. If there are restrictions on publicly sharing data—e.g. participant privacy or use of data from a third party—those must be specified.

Reviewer #1: Yes

4. Is the manuscript presented in an intelligible fashion and written in standard English?

PLOS Mental Health does not copyedit accepted manuscripts, so the language in submitted articles must be clear, correct, and unambiguous. Any typographical or grammatical errors should be corrected at revision, so please note any specific errors here.

Reviewer #1: Yes

5. Review Comments to the Author

Please use the space provided to explain your answers to the questions above. You may also include additional comments for the author, including concerns about dual publication, research ethics, or publication ethics. (Please upload your review as an attachment if it exceeds 20,000 characters)

Reviewer #1: Title: Self-regulation of socioemotional behavior in twin adolescents: structural validation of a multidimensional inventory
Strengths
1. The study is grounded in Pulkkinen’s well-established two-dimensional impulse control model, which integrates cognitive control and behavioral expression. This provides a robust framework for interpreting the findings.
2. The distinction between self-regulation (goal-directed behavior) and self-control (conflict resolution) aligns with contemporary literature (e.g., Inzlicht et al., 2021), adding conceptual clarity.

3. The use of a large, longitudinal twin sample (FinnTwin12) enhances generalizability and allows for genetic and environmental analyses.
4. The Multidimensional Peer Nomination Inventory (MPNI) was adapted for self- and co-twin ratings, demonstrating flexibility in measurement across developmental stages.
5. Factor analyses (including bi-factor modeling) and invariance testing were thorough, supporting the structural validity of the proposed scales (e.g., *p*-factor for psychopathology, src-factor for self-regulation capacity).
6. High Cronbach’s alphas (0.7–0.8) for the *p*-factor and src-factor scales indicate strong internal consistency.
7. Convergent validity was demonstrated via correlations between self- and co-twin ratings, while predictive validity was shown across ages (12–17) and informants (parents, teachers).
8. Twin modeling revealed moderate heritability (~50%) for both *p*-factor and src-factor, consistent with broader behavioral genetics literature (Polderman et al., 2015). The lack of shared environmental effects at ages 14–17 aligns with developmental theories emphasizing unique environmental influences in adolescence.
9. The MPNI’s utility in population-based studies is highlighted, particularly for assessing both strengths (prosocial behavior) and difficulties (hyperactivity, aggression, anxiety).

Limitations and Critiques
1. The omission of depression items (due to separate GBI administration) weakens the *p*-factor’s comprehensiveness, as depression is a core internalizing symptom. The authors acknowledge this but argue that anxiety items partially capture emotional problems.

**Author response:** Yes, we discuss that depression items were not included in the age 17 MPNI in the Methods section on pg 9 (because GBI depression items were collected instead), and it is also discussed on the Limitations paragraph of the Discussion section (pg 30). We believe we have made this point clear.

2. The src-factor’s focus on prosocial behavior neglects socially passive high self-regulation (e.g., introverted compliance). The suggested addition of items like “I keep opinions to myself” is valid but untested.

**Author response:**   Yes, as mentioned in the Discussion, future work should be continued for the study of indicators of compliance in late adolescence/adulthood. The MPNI was developed for children, and the active and passive components of high self-regulation emerged in children. Compliance was assessed at age 12 by the item “Avoids difficult situations by doing something else instead” (Pulkkinen et al., 1999), but at age 17, this item #20 indicated anxiety (low self-regulation and passivity) rather than compliance (high self-regulation and passivity) (Supplementary Table 1), and it was excluded from the MPNI Form SERI. In the factor for Prosocial behavior (Table 1 in the manuscript), item 4 “I am calm and patient” represents compliance (high self-regulation and socially passive), whereas, for instance, item #2 “I am friendly to others” represents constructive behavior (high self-regulation, high activity). The low number of items for compliance (high self-regulation and passive behavior) was not sufficient for the separation of the components of prosocial behavior: constructiveness and compliance, in a factor analysis.

3. The bi-factor model’s interpretation of *p*-factor as “general psychopathology” conflates low self-regulation with psychopathology. While related, these constructs are not identical (e.g., some high-risk behaviors may reflect context-specific dysregulation rather than psychopathology).

**Author response:**   As explained above (see question #1 from editor above), the use of the bi-factor concept was a mistake.

We should have used the concept bipolar. It is now mentioned in the manuscript that the low self-regulation pole of the bipolar factor was expected to indicate vulnerability (an increased risk) for psychopathology (p-factor), whereas the high self-regulation pole of this bipolar factor indicates the capacity for self-regulation (src-factor), which is a novel construct in this study. The hypothesis was based on the long-term longitudinal study (Pulkkinen, 2017) in which low self-regulation in childhood and adolescence predicted many types of problem behaviors (substance use, unemployment, antisocial behavior) until middle age, whereas high self-regulation predicted successful outcomes in studies, occupational area, and human relationships.

4. The src-factor’s single-factor structure (prosocial behavior) lacks differentiation between socially active/passive regulation, unlike the age 11/12 MPNI. This may reflect developmental changes or measurement limitations.

**Author response:**   See Reviewer #1 Limitations/Critique item #2 above.

5. Predictive correlations with adult informants (parents, teachers) were modest (r = 0.09–0.36), suggesting rater-specific biases or developmental shifts. The study does not disentangle these effects.

**Author response:**   There are most likely both informant effects and developmental changes. Socioemotional behavior experienced by a person himself/herself may not be easily observable for teachers and parents. Co-twins have a better opportunity to become aware of them. On the other hand, socioemotional behavior develops during school age. Pulkkinen’s longitudinal study (2017) shows that one’s behavior (e.g., aggression) at age 14 is more predictive of the future than if it was assessed earlier, particularly, if the person had also displayed it earlier; that is consistently at two measurement points.

6. The absence of longitudinal genetic analyses (e.g., cross-lagged twin models) limits insights into how genetic/environmental influences evolve from childhood to adolescence.

**Author response:** We agree that longitudinal analyses would add important context as to how the variation underlying these traits may evolve across development, but they are outside the scope of the current manuscript and would be better suited for a future paper.

7. While measurement invariance was confirmed, sex differences in twin correlations (e.g., higher female MZ correlations for anxiety) were not explored. This could reflect gendered socialization or genetic effects.

**Author response:** Sex limitation models could be an interesting further area of research, it is possible that gendered socialization could be present or there could be sex-specific genetic effects. That said, we believe further sex differences analyses are outside the scope of the current manuscript and would be better suited for a future paper.

8. The sample’s homogeneity (Finnish twins) raises questions about cross-cultural applicability, particularly for prosocial behavior, which may vary by cultural norms.

**Author response:**   The items used for the assessment of high self-regulation covered friendliness, helping behavior, trying to solve difficult problems reasonably, trust, always doing one’s tasks, being calm and patient. They are common indicators of prosocial behavior.

9. The MPNI’s non-clinical focus limits direct comparability to tools like the SDQ, which screens for clinical thresholds. The authors’ suggestion to use aggregate scales (problem/prosocial) in non-clinical samples is pragmatic but untested in clinical populations.

**Author response:**  We have added to the Discussion (p. 28) a reference to Goodman et al. (2010) who have recommended the use of five-factor model in clinical populations but a second-order model in the general population. The results by Staatz et al. (2023) demonstrated that a second-order model (with one first factor for problem behaviors versus prosocial behavior) had a better fit to the data with the general population and validity than the five-factor model.

Recommendations
o Include depression items in future MPNI versions to strengthen the *p*-factor.

**Author response:**  Specific depression items were excluded from the age 17 MPNI because of the use of the GBI test in that data collection stage, but as mentioned in the Discussion (p. 30), some proxy depression items, such as #27 (being excessively sensitive) are included in the anxiety scale of the age 17 MPNI Form SERI. The depression aspect could be strengthened by adding age 12 MPNI depression items: “Worries a lot”, “Seems to be sad and depressed a lot of time” to the MPNI Form SERI (as mentioned in the Discussion).

o Test items capturing socially passive high self-regulation (e.g., emotional suppression) to refine the src-factor.

**Author response:**  New items such as “I keep my opinions to myself rather than express them publicly” (as suggested in Discussion, p. 27) should be created and tested with a sample of young adults/late adolescents.

o Differentiate low self-regulation from psychopathology conceptually (e.g., by linking *p*-factor to specific outcomes like academic failure or substance use).

**Author response:**  As explained above (Reviewer #1 Limitations/Critique item #3 above), it is mentioned in the manuscript that low self-regulation indicates vulnerability (an increased risk) for psychopathology.

o Apply cross-lagged models to examine causal pathways between self-regulation, environment, and outcomes.

**Author response:**   We agree that longitudinal analyses including cross-lagged models would add nuance to our understanding of how these constructs may causally relate to each other, but they are outside the scope of the current manuscript and would be better suited for a future paper.

o Compare genetic/environmental influences across informants (e.g., why teacher ratings show higher shared environment effects).

**Author response:** As noted above in the discussion of cross-informant correlations, genetic analyses across raters could be an interesting future direction, to investigate potential rater-biases or developmental effects. That said, we believe these are outside the scope of the current study.

o Replicate findings in diverse populations to assess cultural validity.

**Author response:** This could be the focus on future work, but it is not in the scope of this study.

o Explore sex-specific genetic/environmental mechanisms (e.g., via sex-limitation models).

**Author response:**   As noted above, we agree sex-limitation models are an interesting future direction, but are outside the scope of the current study.

Conclusion
This study makes significant contributions to the assessment of adolescent socioemotional behavior by validating a theoretically grounded, multidimensional inventory (MPNI SERI). Its strengths include robust factor structure, genetic insights, and developmental validity. However, limitations in measurement breadth, clinical applicability, and cultural generalizability warrant further research. The MPNI shows promise for population-based research but may require adaptations for clinical or cross-cultural use.

6. PLOS authors have the option to publish the peer review history of their article ([what does this mean?](https://track.editorialmanager.com/CL0/https:%2F%2Fjournals.plos.org%2Fmentalthealth%2Fs%2Feditorial-and-peer-review-process%23loc-peer-review-history/1/010f01979a9ebd86-4edd0b63-6ee8-4a46-b8f4-aaa74b1ea0f0-000000/f3hy6LwrXJy6kS1XXOW--4bwivTaZAjaRlx938nwwkg=216)). If published, this will include your full peer review and any attached files.


**Do you want your identity to be public for this peer review?** If you choose “no”, your identity will remain anonymous but your review may still be made public.


For information about this choice, including consent withdrawal, please see our [Privacy Policy](https://track.editorialmanager.com/CL0/https:%2F%2Fwww.plos.org%2Fprivacy-policy/1/010f01979a9ebd86-4edd0b63-6ee8-4a46-b8f4-aaa74b1ea0f0-000000/FQg15HECQbD7EG3oPIUhiKtLd9svWPM4uvnK3nux_Us=216).

Reviewer #1: No
