# Supplementary material for: Self-regulation of socioemotional behavior in twin adolescents: Structural validation of a multidimensional inventory
Source: PLOS Ment Health. 2025 Oct 9;2(10):e0000448. doi: 10.1371/journal.pmen.0000448 (PMC12798259; doi:10.1371/journal.pmen.0000448)
Supplement: Attachment — Submitted filename: Response to Reviewers.docx [file pmen.0000448.s008.docx]

PMEN-D-25-00146R1
Self-regulation of socioemotional behavior in twin adolescents: structural validation of a multidimensional inventory
PLOS Mental Health

Dear Dr. Whipp,

Thank you for submitting your manuscript to PLOS Mental Health. After careful consideration, we feel that it has merit but does not fully meet PLOS Mental Health’s publication criteria as it currently stands. Therefore, we invite you to submit a revised version of the manuscript that addresses the points raised during the review process.

Overall, the previous round of revisions strengthened the study including the structural analyses conducted and I am happy to inform you that, subject to minor revisions, your paper is suitable for publication. The revisions you need to make are as follows:

Table 1. Standardized factor loadings for two and four exploratory factors: There are only two columns for factor loadings under the heading “Two factor model”. It is not clear where are factor loadings for the four-factor model.

Moreover, it is not clear whether Table 1 presents loadings from exploratory or confirmatory factor analysis.

**Author response:** The title for Table 1 has now been modified to “Standardized Factor Loadings from Exploratory Factor Analyses with Two and Four Factors (MPNI Form SERI)”. Additionally, some of Table 1’s columns were not visible before due to the layout of the page, it is now in landscape layout for that page and all of the table can be seen now, apologies. A new “List of Legends for Figures and Tables” file has also now been uploaded.

Please submit your revised manuscript by Oct 07 2025 11:59PM. If you will need more time than this to complete your revisions, please reply to this message or contact the journal office at mentalhealth@plos.org. When you're ready to submit your revision, log on to <https://www.editorialmanager.com/pmen/> and select the 'Submissions Needing Revision' folder to locate your manuscript file.

Please include the following items when submitting your revised manuscript:

- A rebuttal letter that responds to each point raised by the editor and reviewer(s). You should upload this letter as a separate file labeled 'Response to Reviewers'.
- A marked-up copy of your manuscript that highlights changes made to the original version. You should upload this as a separate file labeled 'Revised Manuscript with Track Changes'.
- An unmarked version of your revised paper without tracked changes. You should upload this as a separate file labeled 'Manuscript'.

Guidelines for resubmitting your figure files are available below the reviewer comments at the end of this letter.

We look forward to receiving your revised manuscript.

Kind regards,

Helena R. Slobodskaya, M.D., Ph.D., D.Sc.
Academic Editor
PLOS Mental Health

Journal Requirements:

1. We have noticed that you have uploaded Supporting Information files, but you have not included a list of legends. Please add a full list of legends for your Supporting Information files before or after the references list.

**Author response:** Due to the small adjustment in the Table 1 title, a new “List of Legends for Figures and Tables” file has been uploaded. Additionally, at the end of the manuscript there already was a list of legends, but we did now update the new title for Table 1 there as well. In the Supporting Information file called “Supplemental File” there is a list of legends on the first page for what is contained in that file (these files are also referenced in the main manuscript to tell the reader to go to the supplemental file). At the end of the manuscript I have now added one legend that explains what is in the “Supplemental File”, I hope this satisfies this request.

2. If the reviewer comments include a recommendation to cite specific previously published works, please review and evaluate these publications to determine whether they are relevant and should be cited. There is no requirement to cite these works unless the editor has indicated otherwise.

**Author response:** No citations were added in this revision round.

3. Please review your reference list to ensure that it is complete and correct. If you have cited papers that have been retracted, please include the rationale for doing so in the manuscript text, or remove these references and replace them with relevant current references. Any changes to the reference list should be mentioned in the rebuttal letter that accompanies your revised manuscript. If you need to cite a retracted article, indicate the article’s retracted status in the References list and also include a citation and full reference for the retraction notice.

**Author response:** No retracted papers have been referenced. No references have been adjusted in this revision.

Additional Editor Comments (if provided):


[Note: HTML markup is below. Please do not edit.]

Reviewers' comments:


[NOTE: If reviewer comments were submitted as an attachment file, they will be attached to this email and accessible via the submission site. Please log into your account, locate the manuscript record, and check for the action link "View Attachments". If this link does not appear, there are no attachment files.]

While revising your submission, please upload your figure files to the Preflight Analysis and Conversion Engine (PACE) digital diagnostic tool, <https://pacev2.apexcovantage.com/>. PACE helps ensure that figures meet PLOS requirements. To use PACE, you must first register as a user. Registration is free. Then, login and navigate to the UPLOAD tab, where you will find detailed instructions on how to use the tool. If you encounter any issues or have any questions when using PACE, please email PLOS at figures@plos.org. Please note that Supporting Information files do not need this step.

**Author response:** Figure 1 had not previously been put through the PACE tool, but it has now and a new Figure 1 file has been uploaded.
